# Long-Term Results from an Open-Label Extension Study of Atacicept for the Treatment of IgA Nephropathy

**DOI:** 10.1681/ASN.0000000541

**Published:** 2024-10-26

**Authors:** Jonathan Barratt, Sean Barbour, Robert Brenner, Kerry Cooper, Xuelian Wei, Necmi Eren, Jürgen Floege, Vivekanand Jha, Sung Gyun Kim, Bart Maes, Richard K.S. Phoon, Harmeet Singh, Vladimír Tesař, Richard Lafayette, on behalf of the ORIGIN Phase 2b Investigators

**Affiliations:** 1 College of Medicine Biological Sciences and Psychology, University of Leicester, Leicester, United Kingdom; 2 The University of British Columbia, Vancouver, British Columbia, Canada; 3 Vera Therapeutics, Inc., Brisbane, California; 4 Department of Nephrology, Faculty of Medicine, Kocaeli University, Kocaeli, Turkey; 5 Rheinisch Westfälische Technische Hochschule, Aachen University Hospital, Aachen, Germany; 6 The George Institute for Global Health India, UNSW, New Delhi, India; 7 School of Public Health, Imperial College, London, United Kingdom; 8 Prasanna School of Public Health, Manipal Academy of Higher Education, Manipal, India; 9 Hallym University Sacred Heart Hospital, Anyang, South Korea; 10 AZ Delta, Roeselare, Belgium; 11 The University of Sydney, Sydney, New South Wales, Australia; 12 Western Nephrology, Arvada, Colorado; 13 General University Hospital, Charles University, Prague, Czech Republic; 14 Stanford University, Stanford, California

## Abstract

**Background:**

B-cell activating factor (BAFF) and A proliferation-inducing ligand (APRIL) play key roles in the pathogenesis of IgA nephropathy. Atacicept is a novel fully humanized fusion protein, self-administered at home by subcutaneous injection, that binds and inhibits BAFF and APRIL. By inhibiting BAFF and APRIL, atacicept targets the underlying B-cell–mediated pathogenesis driving disease progression. This study evaluated the long-term efficacy and safety of atacicept in patients with IgA nephropathy over 96 weeks.

**Methods:**

Participants with IgA nephropathy who received atacicept (25, 75, or 150 mg) or placebo in a 36-week phase 2b, randomized, blinded trial were enrolled in an open-label extension study and received atacicept 150 mg for an additional 60 weeks. Key efficacy outcomes were changes in galactose-deficient IgA1 (Gd-IgA1), percentage of participants with hematuria, urine protein-creatinine ratio (UPCR), and eGFR over 96 weeks. Long-term safety data were also evaluated.

**Results:**

There were 113 participants (67 [59%] male; 46 [41%] female) who ranged in age from 18 to 67 years who received ≥1 atacicept dose. Over 96 weeks, safety data demonstrated that atacicept was generally well tolerated. There were also sustained reductions (mean±SEM) in Gd-IgA1 (−66%±2%), percentage of participants with hematuria (−75%; 95% confidence intervals, −87 to −59; in participants with baseline hematuria), and UPCR (−52%±5%). The mean annualized slope of eGFR was −0.6±0.5 ml/min per 1.73 m^2^ through 96 weeks.

**Conclusions:**

Atacicept was also well tolerated over the duration of the study. Atacicept treatment reduced Gd-IgA1, hematuria, and UPCR with stabilization of eGFR through 96 weeks.

**Clinical Trial registry name and registration number:**

Atacicept in Subjects with IgA Nephropathy (ORIGIN 3), NCT04716231.

## Introduction

IgA nephropathy, predominantly diagnosed in young adults, represents a critical challenge in nephrology because of its progressive nature and significant effect on life expectancy and quality.^1–3^ At least 50% of patients with IgA nephropathy develop kidney failure within 10–20 years of initial diagnosis.^4–6^ Although currently available therapies provide benefit, they fail to stop an unrelenting decline in kidney function.^7–9^ Unless the rate of eGFR decline can be minimized, most patients are likely to experience kidney failure within their lifetime despite optimal supportive care and present treatment options.^1–4^ Therefore, a disease-modifying therapy that could stabilize eGFR over the long term would be of great benefit to this patient population.

IgA nephropathy is a disorder of B-cell regulation, with kidney pathology characterized by mesangial IgA-containing immune complex accumulation.^1,10,11^ It is noteworthy that both the pathogenic forms of IgA (commonly measured as galactose-deficient IgA1 [Gd-IgA1]) and the antibodies that recognize Gd-IgA1 are produced by B-lymphocyte−derived plasma cells.^1,10,11^ Therefore, a targeted approach to modulate B-cell activity is mechanistically congruent. The cytokines B-cell activating factor (BAFF) and A proliferation-inducing ligand (APRIL) are members of the TNF family that stimulate the maturation, function, and survival of B cells and plasma cells, including IgA class switching.^10,12,13^ Dysregulation of these cytokines has been associated with elevated serum levels of Gd-IgA1 and Gd-IgA1−specific antibodies, immune complex formation, and recruitment of inflammatory cells to the glomeruli, all central to IgA nephropathy pathogenesis.^10,12,14,15^ Furthermore, BAFF and APRIL levels are elevated in patients with IgA nephropathy and correlate with disease severity.^15,16^

Atacicept is a fully humanized fusion protein composed of the extracellular portion of a native receptor for BAFF and APRIL, transmembrane activator and calcium-modulator and cyclophilin ligand interactor (TACI), with the Fc portion of IgG1, resulting in a soluble receptor for both cytokines with nanomolar potency.^10^ The rational drug design of atacicept enables inhibition of BAFF and APRIL signaling by the B-cell TACI receptor, interrupting the upstream immunopathogenesis of IgA nephropathy.^7–9^ Atacicept received breakthrough therapy designation from the US Food and Drug Administration in May 2024.^17^

Results of the randomized, double-blind, placebo-controlled period of a phase 2b trial of atacicept in participants with IgA nephropathy were previously reported.^10^ The trial met the primary and key secondary end points of mean change in urine protein-creatinine ratio (UPCR) at 24 and 36 weeks, respectively, showing a statistically significant difference with atacicept compared with placebo. Proteinuria has been shown to be an adverse prognostic factor in IgA nephropathy, with a strong relationship between proteinuria and kidney outcomes.^10^ During the 36-week double-blind period, atacicept also demonstrated a statistically significant reduction of serum Gd-IgA1, hematuria resolution, and preservation of eGFR.^18^ All participants who completed the double-blind period on study treatment were eligible for treatment with atacicept during a 60-week, open-label extension (OLE) period. We report the long-term efficacy and safety of atacicept over 96 weeks.

## Methods

### Study Design, Procedures, and Oversight

The Atacicept in Subjects with IgA Nephropathy study design was previously described.^10^ This randomized, international, multicenter, phase 2b trial included a double-blind, placebo-controlled period and OLE period. In the double-blind period, participants received atacicept 25, 75, or 150 mg or placebo, self-administered at home by subcutaneous injection weekly for 36 weeks. Participants who completed the double-blind period were eligible for the OLE, in which all participants received 150 mg of atacicept weekly for 60 weeks.

The study was conducted in accordance with the principles of the Declaration of Helsinki and the International Council for Harmonization of guidelines for Good Clinical Practice. The protocol was approved by the institutional review board or ethics committee at each participating center. All participants provided written informed consent.

The sponsor led the study design, data collection, and analysis with data interpretation informed by the authors. An independent data and safety monitoring committee reviewed the safety data. All authors assisted in data interpretation and writing of the article. The decision to submit the article for publication was made by all the authors and sponsor, which retains the data. The sponsor provided medical writing support.

### Participants

Male or female participants (aged 18 years and older) with biopsy-proven IgA nephropathy within 10 years before screening, 24-hour urine protein >0.75 g/d or UPCR >0.75 mg/mg despite at least 12 weeks on stable doses of maximally tolerated renin-angiotensin-aldosterone system inhibitors (RAASi), and eGFR ≥30 ml/min per 1.73 m^2^ were eligible for enrollment in the double-blind period. Patients on stable-dose sodium-glucose cotransporter-2 inhibitors (SGLT2is) were also eligible. Patients with secondary causes of IgA nephropathy (*e.g*., liver cirrhosis, IgA vasculitis), evidence of rapidly progressive IgA nephropathy (loss of ≥50% of eGFR within 3 months of screening), or evidence of nephrotic syndrome within 6 months of screening (serum albumin >3.0 g/L in association with UPCR >3.5 mg/mg) were excluded.

Demographic data, including age, sex, race/ethnicity, and baseline clinical characteristics, were collected at the time of study enrollment. Participants self-reported demographic information during the initial screening visit. Race and ethnicity categories were collected to ensure the study results are applicable to a broad patient demographic.

### End Point and Assessments

The primary and secondary outcomes of the double-blind period have been previously described.^10^ The key end points of the OLE were change from baseline in Gd-IgA1, resolution of microscopic hematuria evaluated on urine dipstick, change from baseline in UPCR on the basis of 24-hour urine collection, and change from baseline in eGFR. For hematuria, participants with urine dipstick blood ≥1+ at baseline were considered evaluable for resolution defined as urine dipstick blood of trace or negative at 96 weeks. During the OLE, serum Gd-IgA1 was evaluated at 48, 72, and 96 weeks using the KM55 assay,^19^ and 24-hour urine collection for UPCR was performed at 48, 60, 72, 84, and 96 weeks. Urinalysis and eGFR were evaluated at 38, 40, 48, 60, 72, 84, and 96 weeks.

Safety end points included adverse events and clinical laboratory changes, analyzed up to the data cutoff point (June 3, 2024), which included data through the OLE and, for some participants, data during the 26-week safety follow-up period.

### Statistical Analyses

All efficacy analyses were performed using the atacicept-treated population, which consists of all randomized participants, including participants originally randomized to the placebo group, who received ≥1 dose of double-blind or open-label atacicept. All participants in the atacicept-treated population were pooled (atacicept-treated group) to assess the long-term efficacy of atacicept. The baseline for the atacicept-treated population was defined as the double-blind baseline for participants originally randomized to active atacicept groups and as the OLE baseline for participants originally randomized to the placebo group. Thus, participants originally randomized to active atacicept groups received atacicept for up to 96 weeks while participants originally randomized to the placebo group received atacicept for up to 60 weeks.

A mixed-effects model for repeated measures analysis was used to assess the mean percentage change from baseline over time for UPCR and Gd-IgA1. Change from baseline in natural log-transformed data was the dependent variable. Effects for natural log-transformed baseline value, baseline eGFR category, and visit (as a categorical variable) were included as independent fixed effects, and participant was included as a random effect. Analyses were performed on log-transformed data, and results were back-transformed and displayed as percentage change from baseline. In addition, a mixed-effects model with random intercept and random slope was used to assess the annualized total slope in eGFR.

The change from baseline in the percentage of participants with persistent hematuria, *i.e*., urine dipstick blood ≥1, among participants with hematuria at baseline was reported for each postbaseline visit. The 95% confidence intervals for the percentage of participants with persistent hematuria were calculated using the exact Clopper−Pearson method for binomial proportions.

All safety analyses were performed using the safety analysis population, which included all randomized participants who received ≥1 dose of the study drug and were summarized by descriptive statistics.

## Results

### Participant Characteristics

**Figure 1:**
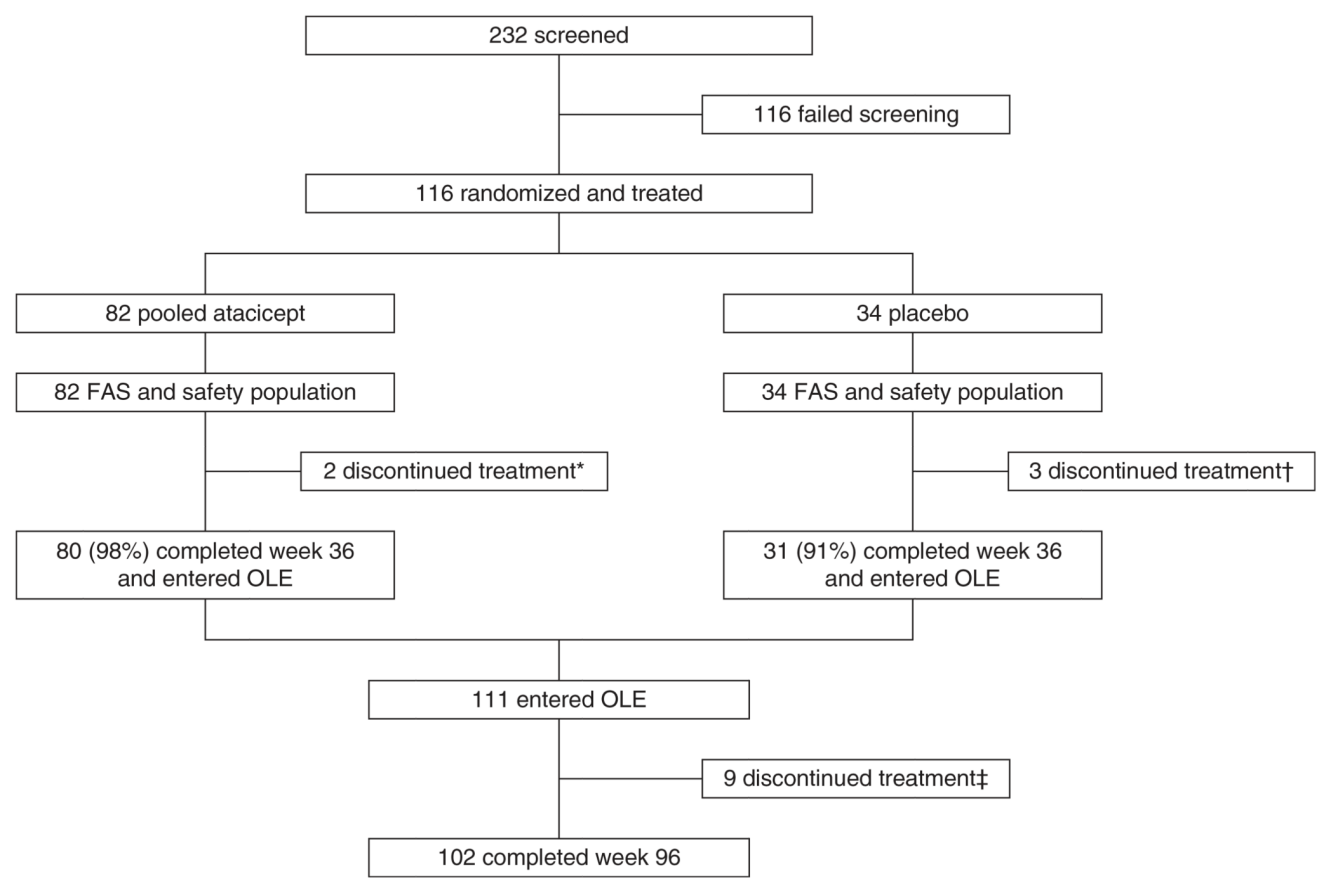
Screening, treatment, and follow-up through week 96. *Discontinued to pursue elective surgery (*n*=1), and discontinued due to positive hepatitis B DNA and adverse event (*n*=1). †Initiated prohibited medication for concomitant disease (*n*=1), discontinued due to plan to start prohibited medication for concomitant disease (*n*=1) and adverse event (*n*=1). ‡Discontinued due to investigator decision (*n*=1), pregnancy (*n*=2), participant withdrawal (*n*=2), surgery (*n*=1), serious adverse event of pneumonia in a heavy smoker, resolved (*n*=1), adverse event of worsening alanine aminotransferase and aspartate aminotransferase (*n*=1), and medical monitor criteria (*n*=1). FAS, full analysis set; OLE, open-label extension.

During the double-blind period, 116 participants were randomized. Eighty-two participants were initially randomized to atacicept and 34 to placebo (31 of 34 received atacicept in this OLE; Figure 1). Among the 113 participants who received ≥1 atacicept dose, 102 (90%) completed treatment in the OLE. Participants were predominantly male (59%) and mainly Asian (45%) or White (52%) with a median (range) age of 37 (18−67) years (Table 1). The mean (±SD) eGFR at baseline was 62±28 ml/min per 1.73 m^2^, and the mean UPCR at baseline was 1.8±1.3 g/g.

### Safety

Through the double-blind and OLE periods, atacicept was well tolerated over a median (range) exposure of 96 weeks (3–99) and a mean of 91 weeks. The safety profile of atacicept in the OLE was similar to that in the doubleblind period (Table 2). The proportion of participants with a treatment-emergent adverse event (TEAE) in the 60-week OLE period (77%) was also similar to that in the atacicept group during the 36-week double-blind period (73%) and to those receiving placebo (82%). The same pattern was seen for drug-related TEAEs. There were 12 serious TEAEs during the OLE period and five during the double-blind period, with three occurring in the placebo group.

In the double-blind period, there were numerically more nonserious infections in the atacicept arm; however, the overall infection rate was balanced between atacicept and placebo-treated participants (Table 2 and Supplemental Table 1). In addition, the frequency and types of infections in atacicept-treated participants were similar during both the double-blind and OLE periods (Supplemental Table 1). There were no opportunistic or fatal infections during the study. Three participants had serious adverse events of infection, there was one participant who had tonsillitis, and two participants had pneumonia, all of which resolved. One participant discontinued the study because of an infection-related adverse event (pneumonia, which resolved).

Serum levels of IgG, IgA, and IgM decreased as expected after atacicept treatment in the randomized portion of the study, consistent with the mechanism of action of B-cell modulation. Serum IgG, IgA, and IgM remained stable during the OLE period after the initial reductions (Supplemental Figure 1). There was one asymptomatic, noninfected participant who discontinued study treatment at week 82 because of IgG level <3 g/L at two consecutive measures at least 4 weeks apart, per protocol. The reductions in Ig were monitored, and no severe infections or adverse events related to immunomodulation were observed during the study.

### Atacicept Treatment and Biomarkers for IgA Nephropathy

**Figure 2:**
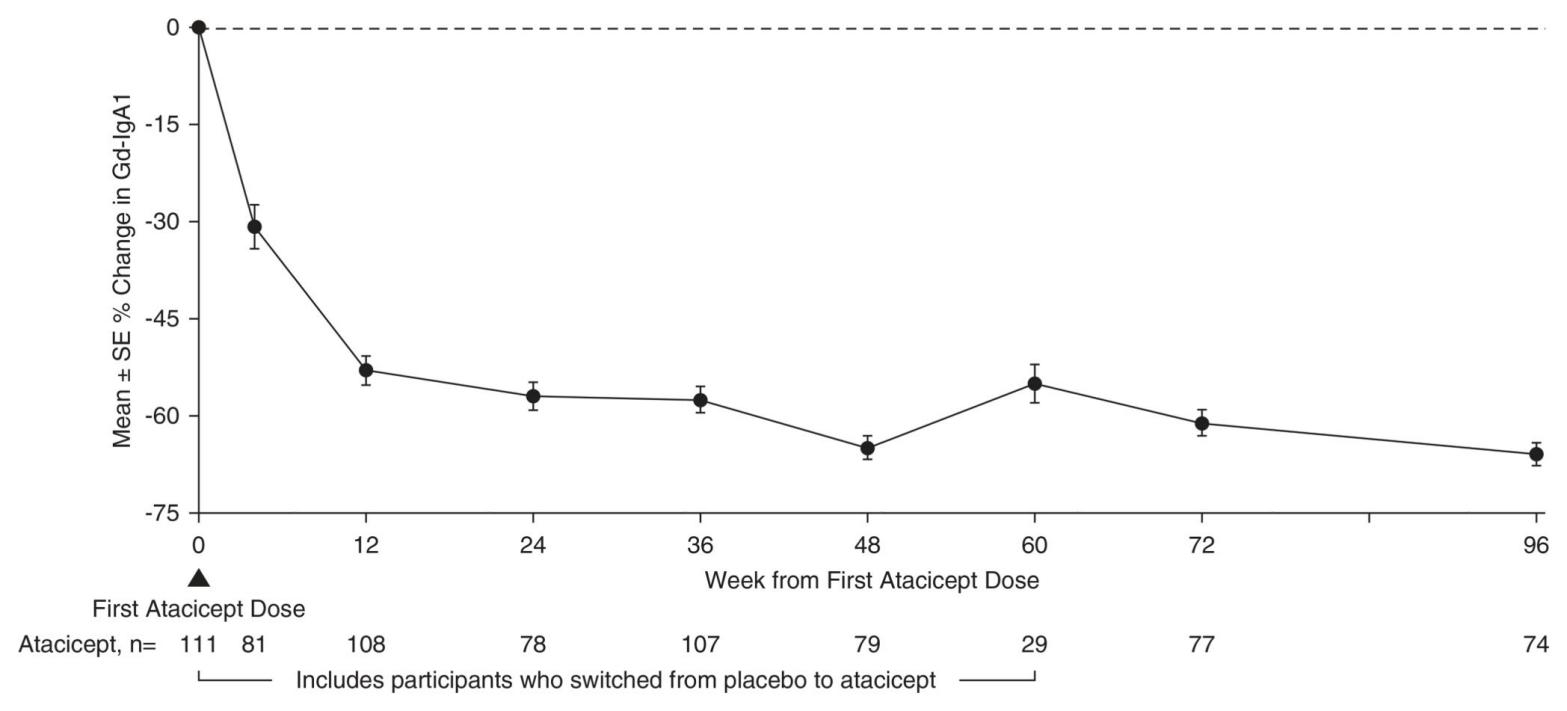
Gd-IgA1 percentage change through 96 weeks percentage changes from baseline were computed using US FDA-endorsed mixed-effects modeling. Atacicept group includes all participants receiving any atacicept dose at any time point, with baseline defined as the last available measurement before the first dose of atacicept. FDA, US Food and Drug Administration; Gd-IgA1, galactose-deficient IgA1.

**Figure 3:**
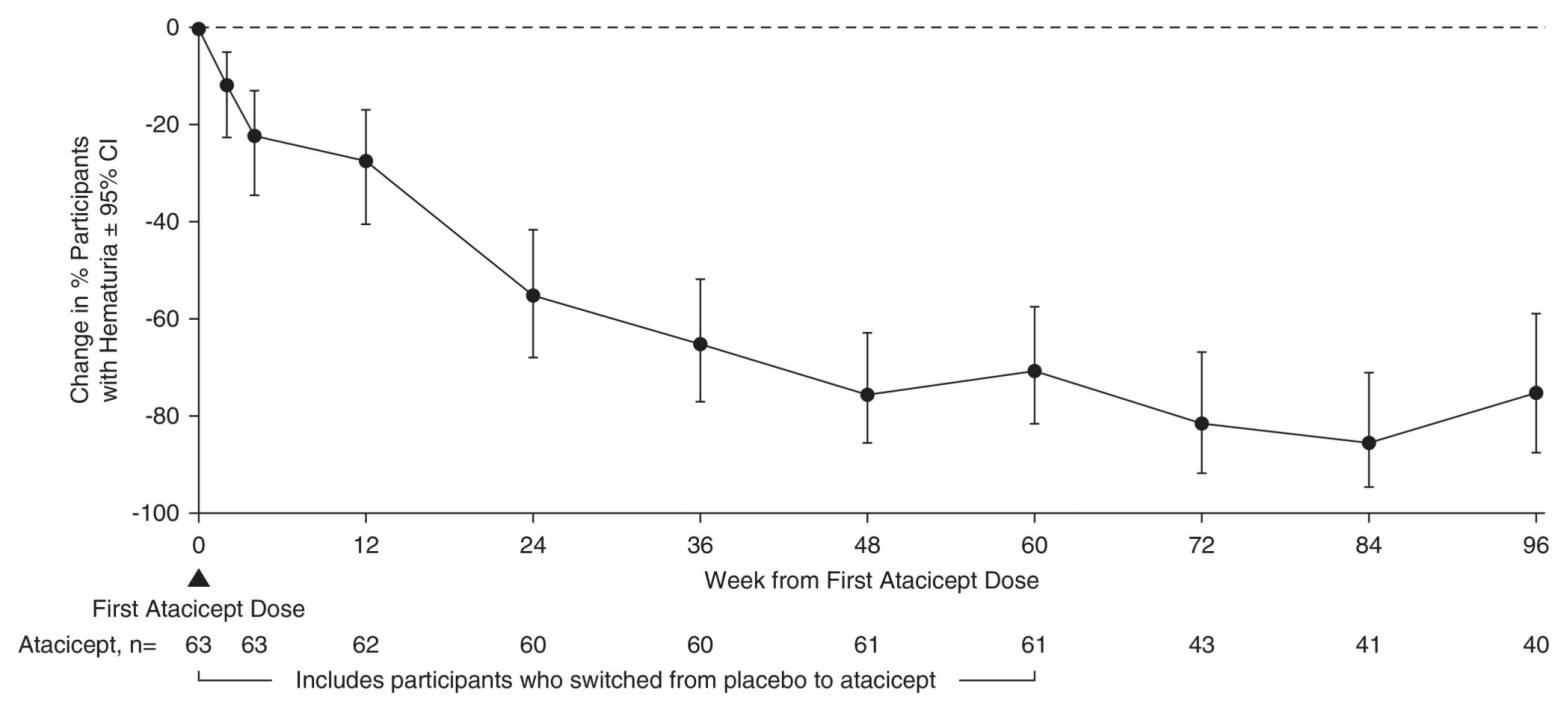
Change in percentage of participants with hematuria through 96 weeks among participants with hematuria at baseline (N563). Percentages represent change from baseline in number of participants with hematuria at each visit (see number of participants with hematuria evaluated at each visit on the lower axis) divided by number with baseline hematuria; microscopic hematuria was evaluated using urine dipstick at a centralized laboratory, and hematuria levels were graded negative/trace, 1+, 2+, or 3+. Atacicept group includes all participants receiving any atacicept dose at any time point, with baseline defined as the last available measurement before the first dose of atacicept. CI, confidence interval.

**Figure 4:**
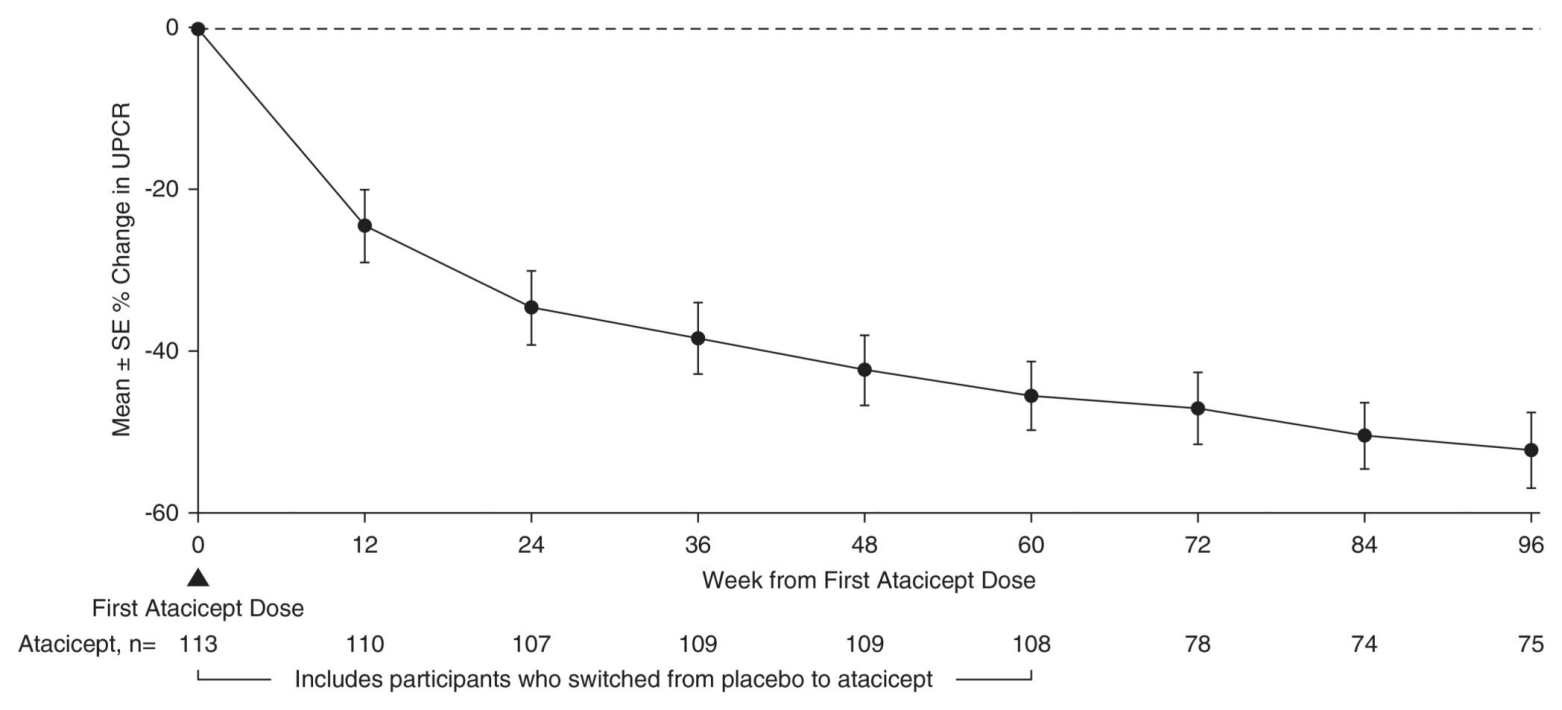
UPCR percentage change through 96 weeks percentage changes from baseline were computed using US FDA-endorsed mixed-effects modeling, which takes into account the effects of baseline UPCR and implicitly imputes missing data as missing at random. Atacicept group includes all participants receiving any atacicept dose at any time point, with baseline defined as the last available measurement before the first dose of atacicept. UPCR, urine protein-creatinine ratio.

**Figure 5:**
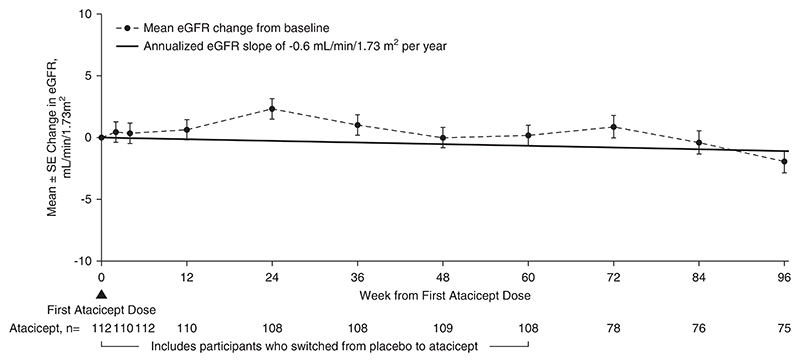
eGFR change through 96 weeks changes from baseline in eGFR (dashed thin line) are analyzed using mixed-model repeated-measures analysis with change from baseline eGFR as the dependent variable; fixed effects for randomized treatment, baseline value, and visit as independent variables; and participant as a random effect. Least squares estimation, SEM, and two-tailed 95% CI are estimated from the model directly. eGFR slope (solid thick line) is analyzed using a mixed-effects model with random intercept and random slope, including eGFR as the dependent variable, baseline value and time as independent variables, and participant and time as random effects. Mean slope and SEM are estimated from the model directly. Atacicept group includes all participants receiving any atacicept dose at any time point, with baseline defined as the last available measurement before the first dose of atacicept.

The 96-week data demonstrated reduction in key biomarkers of disease in participants treated with atacicept. There was a sustained reduction in Gd-IgA1 (mean±SEM) from baseline of −66%±2% (Figure 2). Among participants with hematuria at baseline, the percentage of those with hematuria decreased by −75% (95% confidence interval, −87 to −59) (Figure 3) at 96 weeks. Sustained and consistent reductions in proteinuria were demonstrated throughout the study with a reduction from baseline of −52%±5% at 96 weeks (Figure 4). Treatment with atacicept resulted in a mean eGFR annualized slope of −0.6±0.5 ml/min per 1.73 m^2^ per year (Figure 5).

Participants who received placebo in the double-blind period, when converted to active treatment with atacicept 150 mg at week 36, demonstrated decreases in Gd-IgA1, hematuria, and UPCR similar to what was observed in participants who were initially randomized to atacicept. In addition, conversion to treatment with atacicept was associated with improvement of the eGFR trajectory (Supplemental Table 2).

## Discussion

Presently, at least 50% of patients with IgA nephropathy progress to kidney failure within 10–20 years after diagnosis.^4–6^ The rate of decline in eGFR, despite currently available therapies and the current standard of care, still leaves these patients at substantial risk of kidney failure.^7–9^ The results from this study demonstrate atacicept had a favorable safety profile over 96 weeks of treatment. Importantly, in the three participants who experienced serious infections, all cases resolved without adverse sequelae, and no opportunistic or fatal infections occurred during the study. In addition, participants treated with atacicept, who were at risk of progression despite optimized supportive care with RAASi, achieved a stable eGFR profile over 96 weeks.^20^ Long-term stabilization of eGFR in the OLE period was accompanied by sustained reductions of Gd-IgA1, hematuria, and proteinuria. Collectively, these data demonstrate that B-cell modulation with atacicept may be disease modifying in IgA nephropathy.

Atacicept is a soluble Fc fusion protein using the naturally occurring TACI receptor to target the two cytokines, BAFF and APRIL, that drive B-cell activity, which are key in the pathogenesis of IgA nephropathy.^21^ The dual inhibition of BAFF and APRIL by atacicept modulates B-cell activity, reducing the production of Gd-IgA1 and immune complex formation, thus mitigating glomerular inflammation and injury.^21^ Importantly, this B-cell modulation occurs without evidence of B-cell depletion or immunosuppression, despite stable reductions in Igs, and thus appears amenable to chronic administration.

The conversion of an eGFR profile in patients with IgA nephropathy from one of steady, unrelenting decline to a stable eGFR through 96 weeks is a unique finding and supports the potential of atacicept to modify the natural history of the disease.^20^ While cross-trial effectiveness comparisons should be undertaken with caution because of differences in trial populations, current therapies have not demonstrated this magnitude of effect on eGFR slope. In a subanalysis of the Dapagliflozin and Prevention of Adverse Outcomes in Chronic Kidney Disease trial, patients with IgA nephropathy treated with dapagliflozin, in addition to RAASi, had an eGFR decline less than placebo; nevertheless, treated participants lost kidney function at the rate of 3.5 ml/min per 1.73 m^2^ per year.^7^ Nefecon, recently approved for the treatment of IgA nephropathy, improved UPCR and eGFR outcomes compared with placebo, but participants randomized to Nefecon experienced a decline in eGFR of −6.11 ml/min per 1.73 m^2^ over 2 years.^9^ Treatment with the dual endothelin and angiotensin receptor antagonist, sparsentan, was associated with an eGFR decline of −2.9 ml/min per 1.73 m^2^ per year.^8^ In a phase 2b study, another B-cell modulator, sibeprenlimab, a monoclonal anti-APRIL antibody administered by intravenous infusion in the clinic, resulted in eGFR changes of −2.7, 0.2, and −1.5 ml/min per 1.73 m^2^ at 52 weeks when dosed at 2, 4, and 8 mg/kg, respectively.^22^

On the basis of epidemiologic data from the UK National Registry of Rare Kidney Diseases, which included 2299 adults with IgA nephropathy, almost all patients with eGFR <60 ml/min per 1.73 m^2^ or proteinuria >0.5 g/d were at risk of progressing to kidney failure within their expected lifetime unless the rate of decline in eGFR was maintained, for the rest of their life, at <1 ml/min per 1.73 m^2^ per year.^4^ The rate of eGFR decline observed during treatment with current standard-of-care therapies (RAASi and SGLT2i) and recently approved therapies (*e.g*., Nefecon, sparsentan), exceeds this threshold by some margin. As such, there remains a substantial unmet need to enable patients to minimize their lifetime risk of kidney failure.

Another important finding in this study was the improvement in hematuria seen with atacicept. Not all participants had hematuria at baseline, but hematuria resolution was seen in 75% of participants treated with atacicept at 96 weeks. Hematuria is believed to be a manifestation of glomerular inflammation in IgA nephropathy.^23^ Atacicept-induced reductions in Gd-IgA1, associated antibodies, and immune complex formation are likely to directly reduce the glomerular damage that results in hematuria. The time course of reductions in hematuria and proteinuria suggests that atacicept compares favorably with the anti-inflammatory effects of systemic corticosteroids and complement inhibitors.^9^ This effect is likely to be due to a rapid decline in pathogenic immune complex levels, alongside a possible direct effect of reducing BAFF and APRIL stimulation of innate immune cells, such as macrophages, which also express the TACI receptor.^24^

One of the strengths of the study is that the population is representative of the overall IgA nephropathy population. Similar to the UK National Registry of Rare Kidney Diseases cohort,^4^ the population in this study is representative of patients with IgA nephropathy who are at risk of disease progression with a mean baseline UPCR of 1.8 g/g and a median eGFR of 56 ml/min per 1.73 m^2^. The results are, therefore, likely to be applicable to the real-world IgA nephropathy population at risk of disease progression. In addition, there was a high rate of participant retention in the OLE period. However, there are important limitations to the study, including the relatively small study size, lack of repeated kidney biopsy data, and small percentage (*n*=15, 13% at baseline) of participants on SGLT2is, which have now become part of the standard of care for CKD management. The study design allowed for an OLE period, but this also meant there was no placebo comparison beyond 36 weeks, and long-term eGFR results are descriptive in nature. Finally, a standard for quantifying hematuria is a manual red blood cell count by an experienced operator or an automated red blood cell count; however, these are not practical in a multicenter, global trial.

The study demonstrates the potential for the B-cell modulator, atacicept, to address the underlying pathogenesis of IgA nephropathy by binding BAFF and APRIL, resulting in reductions in pathogenic forms of IgA, hematuria, and proteinuria and a stable eGFR profile. These findings support the ongoing, pivotal phase 3 trial, Atacicept in Subjects with IgA Nephropathy 3 (NCT04716231), and demonstrate that atacicept offers a potentially safe, long-term, disease-modifying treatment option for IgA nephropathy.

## Supplementary Material

Supplementary Material

## Figures and Tables

**Table 1:** Demographic and baseline characteristics

COLUMN HEADING	Atacicept Treatment^a^ (n=113)
Age, yr, median (range)	37 (18–67)
**Sex, *n* (%)**	
Male	67 (59)
Female	46 (41)
**Race, n (%)**	
Asian	51 (45)
Native Hawaiian or Other Pacific Islander	1 (1)
Not reported	2 (2)
White	59 (52)
**Ethnicity**, *n* (%)	
Hispanic or Latino	4 (4)
Not Hispanic or Latino	108 (96)
Unknown	1 (1)
**Hematuria**, *n* (%)	
Negative/trace	49 (43)
1 + or higher	63 (56)
Missing	1 (1)
**BP, mm Hg, mean (SD)**	
Systolic	127 (14)
Diastolic	80 (10)
**24-h UPCR, g/g**	
Mean (SD)	1.8 (1.3)
Median (IQR)	1.4 (1.0–2.2)
UACR_b_ g/g, mean (SD)	1.3 (0.9)
**eGFR,**^b^ **ml/min per 1.73 m**^**2**^	
Mean (SD)	62 (28)
Median (IQR)	56 (41–73)
**eGFR category, ml/min per 1.73 m** ^**2**^ **, n (%)**	
<30	6 (5)
≥30 to <45	31 (27)
≥45	75 (66)
Gd-IgA1, *μ*g/L, mean (SD)	5785 (3221)
IgA, mg/dl, mean (SD)	311 (115)
IgG, mg/dl, mean (SD)	1099 (247)
IgM, mg/dl, mean (SD)	104 (69)
**Use of RAASi**, *n* (%)	
No RAASi	7 (6)
ACEi only	31 (27)
ARB only	66 (58)
Dual RAASi^c^	9 (8)
**Use of SGLT2i**, *n* (%)	
Yes	15 (13)
No	98 (87)

ACEi, angiotensin-converting enzyme inhibitor; ARB, angiotensin II receptor blocker; Gd-IgA1, galactose-deficient IgA1; IQR, interquartile range; MRA, mineralocorticoid receptor antagonist; RAASi, renin-angiotensin-aldosterone system inhibitor; SGLT2i, sodium-glucose cotransporter-2 inhibitor; UACR, urine albumin-creatinine ratio; UPCR, urine protein-creatinine ratio.

a Atacicept group includes all participants receiving any atacicept dose at any time point, with baseline defined as the last available measurement before the first dose of atacicept.

b eGFR was determined using the CKD Epidemiology formula.

c Included participants using a stable regimen of angiotensin-converting enzyme inhibitor+angiotensin II receptor blocker, angiotensin-converting enzyme inhibitor+mineralocorticoid receptor antagonist, or angiotensin II receptor blocker+mineralocorticoid receptor antagonist.

**Table 2:** Overall safety

Participants, *n* (%)	Double-Blind Baseline to Week 36			OLE Week 36-96^a^
	Placebo (*n*=34)	All Atacicept (*n*=82)		Atacicept 150 mg (*n*=111)
TEAEs	28 (82)	60 (73)		85 (77)
Infections and infestations	11 (32)	35 (43)		43 (39)
Study drug-related TEAEs^b^	14 (41)	42 (51)		52 (47)
Serious TEAEs^c^	3 (9)	2 (2)		12 (11)
TEAEs leading to study drug discontinuation^d^	1 (3)	1 (1)		2 (2)
Deaths	0	0		0

AEs, adverse events; OLE, open-label extension; TEAE, treatment-emergent adverse event.

a Week 96 cutoff includes all safety data as of June 3, 2024, including visits past week 96. Adverse events were considered treatment emergent during the open-label extension period if they started after the first dose of open-label atacicept 150 mg through the end of the study. This group represents the 80 participants randomized to atacicept and the 31 participants randomized to placebo who entered the open-label extension period.

b Most of the study drug-related treatment-emergent adverse events were injection site reactions and one contributed to drug discontinuation during the double-blind period.

c Double-blind placebo: anaphylactic reaction due to a peanut allergy (*n*=1), forearm fracture (*n*=1), and flank pain and ulnar nerve paralysis (*n*=1; both reported in the same participant); double-blind atacicept: multiple fractures (*n*=1, atacicept 75 mg) and norovirus gastroenteritis (*n*=1, atacicept 150 mg); open-label extension: excess abdominal fat and left basal bronchopneumonia (*n*=1), AKI (*n*=1), angioedema (*n*=1), termination of pregnancy (*n*=1), postcricoid ulcer (*n*=1), pancreatitis, passed-out common bile duct stone, and acute cholecystitis (*n*=1), tonsillitis (*n*=1), pneumonia (*n*=1), acute coronary syndrome required hospitalization (*n*=1), left fifth metatarsophalangeal joint gout (*n*=1), mild flare of IgA nephropathy (*n*=1), and urethral stricture worsening (*n*=1).

d Double-blind placebo: discontinuation due to worsening flank pain that was not resolved unrelated to study treatment (*n*=1); double-blind atacicept: discontinuation due to injection site reaction and positive hepatitis B DNA that resolved without treatment 3 weeks later, normal liver function throughout (*n*=1); open-label extension: discontinuation due to pneumonia in a heavy smoker, resolved (*n*=1); and worsening alanine aminotransferase and aspartate aminotransferase, resolved and unrelated to study treatment (*n*=1).

## Data Availability

Partial restrictions to the data and/or materials apply. Vera Therapeutics shares anonymized individual patient data upon request or as required by law or regulation with qualified external researchers based on submitted curriculum vitae, ensuring there is no conflict of interest. The request proposal must also include a statistician. Approval of such requests is at Vera’s discretion and is dependent on the nature of the request, the merit of the research proposed, the availability of the data, and the intended use of the data.

## Key Points

- Participants who completed a 36-week double-blind study of atacicept were eligible for a 60-week, open-label extension study.
- Atacicept 96-week treatment resulted in sustained reductions in galactose-deficient IgA1, hematuria, and urine protein-creatinine ratio.
- The slope of the eGFR was similar to that observed in the general population without kidney disease.
