# Supplementary material for: Long-Term Results from an Open-Label Extension Study of Atacicept for the Treatment of IgA Nephropathy
Source: J Am Soc Nephrol. 2024 Oct 26;36(4):679–87. doi: 10.1681/ASN.0000000541 (PMC7616790; doi:10.1681/ASN.0000000541)
Supplement: Supplementary file 1 [file jasn-36-679-s001.pdf]

ASN
American Society of Nephrology
JASN
Journal of the American Society of Nephrology
KIDNEY360
Accessing Our World From Every Angle
CJASN
Clinical Journal of the American Society of Nephrology

ASN Journal Disclosure Form

As per ASN journal policy, I have disclosed any financial relationships or commitments I have held in the past 36 months as included below. I have listed my Current Employer below to indicate there is a relationship requiring disclosure. If no relationship exists, my Current Employer is not listed.

S. Barbour reports the following:
Employer: University of British Columbia; Consultancy: Visterra, Achillion, Alexion, Novartis, Vera, Pfizer, Eledon, HIBio, BeiGene, BioCryst, Roche; Research Funding: Roche, Alexion, Novartis; and Honoraria: Kirin.

I understand that the information above will be published within the journal article, if accepted, and that failure to comply and/or to accurately and completely report the potential financial conflicts of interest could lead to the following: 1) Prior to publication, article rejection, or 2) Post-publication, sanctions ranging from, but not limited to, issuing a correction, reporting the inaccurate information to the authors' institution, banning authors from submitting work to ASN journals for varying lengths of time, and/or retraction of the published work.

Name: Sean Barbour
Manuscript ID: JASN-2024-001198
Manuscript Title: LBCT: Long-term Results From an Open-label Extension Study of Atacicept for the Treatment of IgA Nephropathy,
Date of Completion: September 20, 2024
Disclosure Updated Date: April 16, 2024

ASN
American Society of Nephrology
JASN
Journal of the American Society of Nephrology
KIDNEY360
Accessing Our World From Every Angle
CJASN
Clinical Journal of the American Society of Nephrology

ASN Journal Disclosure Form

As per ASN journal policy, I have disclosed any financial relationships or commitments I have held in the past 36 months as included below. I have listed my Current Employer below to indicate there is a relationship requiring disclosure. If no relationship exists, my Current Employer is not listed.

J. Barratt reports the following:
Employer: UNIVERSITY OF LEICESTER; Consultancy: Alexion, Astellas, Alebund, Alnylam, Alpine, Argenx, BioCryst, Calliditas, Chinook, Dimerix, HiBio, Kira, Novartis, Omeros, Otsuka, Travere Therapeutics, Q32 Bio, Roche, Sanofi, Takeda, Vera Therapeutics, Vifor, Visterra; Research Funding: Alexion, Novartis; GlaxoSmithKline; Calliditas, Visterra, Chinook, Omeros, Galapagos, argenx, Travere Therapeutics; and Advisory or Leadership Role: Editorial Board of Kidney International, CJASN, Glomerular Diseases & Clinical Science; Treasurer International IgA Nephropathy Network.

I understand that the information above will be published within the journal article, if accepted, and that failure to comply and/or to accurately and completely report the potential financial conflicts of interest could lead to the following: 1) Prior to publication, article rejection, or 2) Post-publication, sanctions ranging from, but not limited to, issuing a correction, reporting the inaccurate information to the authors' institution, banning authors from submitting work to ASN journals for varying lengths of time, and/or retraction of the published work.

Name: Jonathan Barratt
Manuscript ID: JASN-2024-001198
Manuscript Title: LBCT: Long-term Results From an Open-label Extension Study of Atacicept for the Treatment of IgA Nephropathy
Date of Completion: September 16, 2024
Disclosure Updated Date: August 28, 2024

ASN American Society of Nephrology
JASN Journal of the American Society of Nephrology
KIDNEY360 Accessing Our World From Every Angle
CJASN Clinical Journal of the American Society of Nephrology

ASN Journal Disclosure Form

As per ASN journal policy, I have disclosed any financial relationships or commitments I have held in the past 36 months as included below. I have listed my Current Employer below to indicate there is a relationship requiring disclosure. If no relationship exists, my Current Employer is not listed.

R. Brenner reports the following:
Employer: Vera Therapeutics; Ownership Interest: Vera Therapeutics; and Advisory or Leadership Role: Vera Therapeutics corporate officer.

I understand that the information above will be published within the journal article, if accepted, and that failure to comply and/or to accurately and completely report the potential financial conflicts of interest could lead to the following: 1) Prior to publication, article rejection, or 2) Post-publication, sanctions ranging from, but not limited to, issuing a correction, reporting the inaccurate information to the authors' institution, banning authors from submitting work to ASN journals for varying lengths of time, and/or retraction of the published work.

Name: Robert M. Brenner
Manuscript ID: JASN-2024-001198
Manuscript Title: LBCT: Long-term results from an open label extension study of Atacicept for the treatment of IgA Nephropathy
Date of Completion: September 20, 2024
Disclosure Updated Date: September 20, 2024

ASN American Society of Nephrology | JASN Journal of the American Society of Nephrology | KIDNEY360 Accessing Our World From Every Angle | CJASN Clinical Journal of the American Society of Nephrology

ASN Journal Disclosure Form

As per ASN journal policy, I have disclosed any financial relationships or commitments I have held in the past 36 months as included below. I have listed my Current Employer below to indicate there is a relationship requiring disclosure. If no relationship exists, my Current Employer is not listed.

K. Cooper reports the following:
Employer: Vera Therapeutics; and Ownership Interest: Vera Therapeutics.

I understand that the information above will be published within the journal article, if accepted, and that failure to comply and/or to accurately and completely report the potential financial conflicts of interest could lead to the following: 1) Prior to publication, article rejection, or 2) Post-publication, sanctions ranging from, but not limited to, issuing a correction, reporting the inaccurate information to the authors' institution, banning authors from submitting work to ASN journals for varying lengths of time, and/or retraction of the published work.

Name: Kerry Cooper
Manuscript ID: JASN-2024-001198
Manuscript Title: Long-Term Results from an Open-Label Extension Study of Atacicept for the Treatment of IgA Nephopathy
Date of Completion: September 15, 2024
Disclosure Updated Date: September 15, 2024

ASN American Society of Nephrology
JASN Journal of the American Society of Nephrology
KIDNEY360 Accessing Our World From Every Angle
CJASN Clinical Journal of the American Society of Nephrology

ASN Journal Disclosure Form

As per ASN journal policy, I have disclosed any financial relationships or commitments I have held in the past 36 months as included below. I have listed my Current Employer below to indicate there is a relationship requiring disclosure. If no relationship exists, my Current Employer is not listed.

N. Eren reports the following:
Employer: Kocaeli University; and Honoraria: Astra Zeneca, Sanofi, Takeda, Abdi ?brahim Otsuka.

I understand that the information above will be published within the journal article, if accepted, and that failure to comply and/or to accurately and completely report the potential financial conflicts of interest could lead to the following: 1) Prior to publication, article rejection, or 2) Post-publication, sanctions ranging from, but not limited to, issuing a correction, reporting the inaccurate information to the authors' institution, banning authors from submitting work to ASN journals for varying lengths of time, and/or retraction of the published work.

Name: Necmi Eren
Manuscript ID: JASN-2024-001198R1
Manuscript Title: Long-term Results From an Open-label Extension Study of Atacicept for the Treatment of IgA Nephropathy.
Date of Completion: October 5, 2024
Disclosure Updated Date: May 22, 2024

ASN American Society of Nephrology | JASN Journal of the American Society of Nephrology | KIDNEY360 Accessing Our World From Every Angle | CJASN Clinical Journal of the American Society of Nephrology

ASN Journal Disclosure Form

As per ASN journal policy, I have disclosed any financial relationships or commitments I have held in the past 36 months as included below. I have listed my Current Employer below to indicate there is a relationship requiring disclosure. If no relationship exists, my Current Employer is not listed.

J. Floege reports the following:
Consultancy: AstraZeneca, Bayer, Boehringer, Calliditas, CSL Vifor, HiBioNovo Nordisk, Novartis, Omeros, Otsuka, Roche, Stada, Travere, Vifor, Vera Tx, Visterra; Honoraria: AstraZeneca, Bayer, Boehringer, Calliditas, CSL Vifor, HiBioNovo Nordisk, Novartis, Omeros, Otsuka, Roche, Stada, Travere, Vifor, Vera Tx, Visterra; and Speakers Bureau: AstraZeneca, Bayer, Boehringer, Calliditas, CSL Vifor, HiBioNovo Nordisk, Novartis, Omeros, Otsuka, Roche, Stada, Travere, Vifor, Vera Tx, Visterra.

I understand that the information above will be published within the journal article, if accepted, and that failure to comply and/or to accurately and completely report the potential financial conflicts of interest could lead to the following: 1) Prior to publication, article rejection, or 2) Post-publication, sanctions ranging from, but not limited to, issuing a correction, reporting the inaccurate information to the authors' institution, banning authors from submitting work to ASN journals for varying lengths of time, and/or retraction of the published work.

Name: Jürgen Floege
Manuscript ID: JASN-2024-001198R1
Manuscript Title: Long-Term Results from an Open-label Extension Study of Atacicept for the Treatment of IgA Nephropathy
Date of Completion: October 6, 2024
Disclosure Updated Date: July 12, 2024

ASN American Society of Nephrology · JASN Journal of the American Society of Nephrology · KIDNEY360 Accessing Our World From Every Angle · CJASN Clinical Journal of the American Society of Nephrology

ASN Journal Disclosure Form

As per ASN journal policy, I have disclosed any financial relationships or commitments I have held in the past 36 months as included below. I have listed my Current Employer below to indicate there is a relationship requiring disclosure. If no relationship exists, my Current Employer is not listed.

V. Jha reports the following:
Employer: George Institute for Global Health India; and Honoraria: Baxter Healthcare; Bayer; Zydus Cadilla; Travere, Vera, Visterra, Astra Zeneca, Boehringer Ingelheim, Vera, Biocryst, Chinook.

I understand that the information above will be published within the journal article, if accepted, and that failure to comply and/or to accurately and completely report the potential financial conflicts of interest could lead to the following: 1) Prior to publication, article rejection, or 2) Post-publication, sanctions ranging from, but not limited to, issuing a correction, reporting the inaccurate information to the authors' institution, banning authors from submitting work to ASN journals for varying lengths of time, and/or retraction of the published work.

Name: Vivekanand Jha
Manuscript ID: JASN-2024-001198
Manuscript Title: LBCT: Long-term Results From an Open-label Extension Study of Atacicept for the Treatment of IgA Nephropathy
Date of Completion: September 18, 2024
Disclosure Updated Date: May 22, 2024

ASN
American Society of Nephrology
JASN
Journal of the American Society of Nephrology
KIDNEY360
Accessing Our World From Every Angle
CJASN
Clinical Journal of the American Society of Nephrology

ASN Journal Disclosure Form

As per ASN journal policy, I have disclosed any financial relationships or commitments I have held in the past 36 months as included below. I have listed my Current Employer below to indicate there is a relationship requiring disclosure. If no relationship exists, my Current Employer is not listed.

S. Kim reports the following:
Employer: Hallym University Sacred Heart Hospital; Consultancy: Alpine; AstraZeneca; Billy; GSK; Alexion; Bayer; Research Funding: Fibrogen; GSK; JW; VALOR; Alexion; Roche; Bayer;; Honoraria: GSK; bayer; Alpine; Alexion; and Advisory or Leadership Role: KSN.

I understand that the information above will be published within the journal article, if accepted, and that failure to comply and/or to accurately and completely report the potential financial conflicts of interest could lead to the following: 1) Prior to publication, article rejection, or 2) Post-publication, sanctions ranging from, but not limited to, issuing a correction, reporting the inaccurate information to the authors' institution, banning authors from submitting work to ASN journals for varying lengths of time, and/or retraction of the published work.

Name: Sung Gyun Kim
Manuscript ID: JASN-2024-001198
Manuscript Title: LBCT: Long-term Results From an Open-label Extension Study of Atacicept for the Treatment of IgA Nephropathy
Date of Completion: September 13, 2024
Disclosure Updated Date: September 13, 2024

ASN American Society of Nephrology | JASN Journal of the American Society of Nephrology | KIDNEY360 Accessing Our World From Every Angle | CJASN Clinical Journal of the American Society of Nephrology

ASN Journal Disclosure Form

As per ASN journal policy, I have disclosed any financial relationships or commitments I have held in the past 36 months as included below. I have listed my Current Employer below to indicate there is a relationship requiring disclosure. If no relationship exists, my Current Employer is not listed.

R. Lafayette reports the following:
Employer: Stanford University; Consultancy: Calliditas, Inc; Chinook, Inc: Omeros, Inc; Otsuka, Inc. ; Alexion, Inc, Travere, Inc., Vera, inc. , Novartis, Aurinia; Visterra, Alpine Bio, Beigene.; and Research Funding: NIH, Pfizer, Roche, Amgen, Otsuka, Omeros, Calliditas, Travere, Apellis, Chinook, Vera, Beigene, .

I understand that the information above will be published within the journal article, if accepted, and that failure to comply and/or to accurately and completely report the potential financial conflicts of interest could lead to the following: 1) Prior to publication, article rejection, or 2) Post-publication, sanctions ranging from, but not limited to, issuing a correction, reporting the inaccurate information to the authors' institution, banning authors from submitting work to ASN journals for varying lengths of time, and/or retraction of the published work.

Name: Richard A. Lafayette
Manuscript ID: (JASN-2024-001198) and Manuscript Title ("LBCT: Long-term Results From an Open-label Extension Study of Atacicept for the Treatment of IgA Nephropathy
Manuscript Title: LBCT: Long-term Results From an Open-label Extension Study of Atacicept for the Treatment of IgA Nephropathy
Date of Completion: September 13, 2024
Disclosure Updated Date: May 1, 2024

ASN American Society of Nephrology | JASN Journal of the American Society of Nephrology | KIDNEY360 Accessing Our World From Every Angle | CJASN Clinical Journal of the American Society of Nephrology

ASN Journal Disclosure Form

As per ASN journal policy, I have disclosed any financial relationships or commitments I have held in the past 36 months as included below. I have listed my Current Employer below to indicate there is a relationship requiring disclosure. If no relationship exists, my Current Employer is not listed.

B. Maes reports the following:
Employer: AZ Delta Roeselare ; Deltalaan 1; 8800 Roeselare; Belgium; and Advisory or Leadership Role: Novartis; GSK; VeraTherapeutics.

I understand that the information above will be published within the journal article, if accepted, and that failure to comply and/or to accurately and completely report the potential financial conflicts of interest could lead to the following: 1) Prior to publication, article rejection, or 2) Post-publication, sanctions ranging from, but not limited to, issuing a correction, reporting the inaccurate information to the authors' institution, banning authors from submitting work to ASN journals for varying lengths of time, and/or retraction of the published work.

Name: Bart D. Maes
Manuscript ID: JASN-2024-001198
Manuscript Title: LBCT: Long-term Results From an Open-label Extension Study of Atacicept for the Treatment of IgA Nephropathy
Date of Completion: September 16, 2024
Disclosure Updated Date: September 16, 2024

ASN American Society of Nephrology
JASN Journal of the American Society of Nephrology
KIDNEY360 Accessing Our World From Every Angle
CJASN Clinical Journal of the American Society of Nephrology

ASN Journal Disclosure Form

As per ASN journal policy, I have disclosed any financial relationships or commitments I have held in the past 36 months as included below. I have listed my Current Employer below to indicate there is a relationship requiring disclosure. If no relationship exists, my Current Employer is not listed.

R. Phoon reports the following:
Employer: Westmead Hospital; Consultancy: AstraZeneca; Honoraria: AstraZeneca, Boehringer Ingelheim - Lilly, Pharmaceutical Society of Australia, Reed Medical; Advisory or Leadership Role: Advisory Board, AstraZeneca (paid); Board member (Treasurer and Secretary), Ballet Without Borders (unpaid); Councillor, The Hospitals Contribution Fund of Australia (unpaid); and Speakers Bureau: AstraZeneca, Boehringer Ingelheim - Lilly.

I understand that the information above will be published within the journal article, if accepted, and that failure to comply and/or to accurately and completely report the potential financial conflicts of interest could lead to the following: 1) Prior to publication, article rejection, or 2) Post-publication, sanctions ranging from, but not limited to, issuing a correction, reporting the inaccurate information to the authors' institution, banning authors from submitting work to ASN journals for varying lengths of time, and/or retraction of the published work.

Name: Richard K S Phoon
Manuscript ID: JASN-2024-001198
Manuscript Title: LBCT: Long-term Results From an Open-label Extension Study of Atacicept for the Treatment of IgA Nephropathy
Date of Completion: September 25, 2024
Disclosure Updated Date: August 26, 2024

ASN American Society of Nephrology | JASN Journal of the American Society of Nephrology | KIDNEY360 Accessing Our World From Every Angle | CJASN Clinical Journal of the American Society of Nephrology

ASN Journal Disclosure Form

As per ASN journal policy, I have disclosed any financial relationships or commitments I have held in the past 36 months as included below. I have listed my Current Employer below to indicate there is a relationship requiring disclosure. If no relationship exists, my Current Employer is not listed.

H. Singh reports the following:
Employer: Western Nephrology, Parexel corporation; Consultancy: one KOL meeting : travere; Ownership Interest: astra zeneca, medtronics, uhc, alnylam, cigna, cvs,; Research Funding: i have been a Site PI for many pharmaceutical clinical trials.; Vera, Akebia, Reata, cloudcath, Alpine, astra zeneca, Cara, Natera, Calliditas; and Honoraria: travere: attended KOL meeting.

I understand that the information above will be published within the journal article, if accepted, and that failure to comply and/or to accurately and completely report the potential financial conflicts of interest could lead to the following: 1) Prior to publication, article rejection, or 2) Post-publication, sanctions ranging from, but not limited to, issuing a correction, reporting the inaccurate information to the authors' institution, banning authors from submitting work to ASN journals for varying lengths of time, and/or retraction of the published work.

Name: Harmeet Singh
Manuscript ID: JASN-2024-001198
Manuscript Title: "LBCT: Long-term Results From an Open-label Extension Study of Atacicept for the Treatment of IgA Nephropathy,
Date of Completion: September 17, 2024
Disclosure Updated Date: August 25, 2024

ASN American Society of Nephrology · JASN Journal of the American Society of Nephrology · KIDNEY360 Accessing Our World From Every Angle · CJASN Clinical Journal of the American Society of Nephrology

ASN Journal Disclosure Form

As per ASN journal policy, I have disclosed any financial relationships or commitments I have held in the past 36 months as included below. I have listed my Current Employer below to indicate there is a relationship requiring disclosure. If no relationship exists, my Current Employer is not listed.

V. Tesar reports the following:
Consultancy: AstraZeneca, Bayer, Boehringer-Ingelheim, Calliditas, GSK, Eli Lilly, Novartis, Otsuka, Travere, Vera; Honoraria: For consultancy as follows: AstraZeneca, Bayer, Boehringer-Ingelheim, Calliditas, Eli Lilly, Novartis, Travere, Vera; and Advisory or Leadership Role: member of the of the steering committee of clinical trials sponsored by Calliditas, Novartis, Otsuka, Travere, Vera.

I understand that the information above will be published within the journal article, if accepted, and that failure to comply and/or to accurately and completely report the potential financial conflicts of interest could lead to the following: 1) Prior to publication, article rejection, or 2) Post-publication, sanctions ranging from, but not limited to, issuing a correction, reporting the inaccurate information to the authors' institution, banning authors from submitting work to ASN journals for varying lengths of time, and/or retraction of the published work.

Name: Vladimir Tesar
Manuscript ID: JASN-2024-001198
Manuscript Title: LBCT: Long-term Results From an Open-label Extension Study of Atacicept for the Treatment of IgA Nephropathy,
Date of Completion: September 18, 2024
Disclosure Updated Date: May 8, 2024

ASN American Society of Nephrology | JASN Journal of the American Society of Nephrology | KIDNEY360 Accessing Our World From Every Angle | CJASN Clinical Journal of the American Society of Nephrology

ASN Journal Disclosure Form

As per ASN journal policy, I have disclosed any financial relationships or commitments I have held in the past 36 months as included below. I have listed my Current Employer below to indicate there is a relationship requiring disclosure. If no relationship exists, my Current Employer is not listed.

X. Wei reports the following:
Employer: Vera Therapeutics; and Ownership Interest: Vera Therapeutics.

I understand that the information above will be published within the journal article, if accepted, and that failure to comply and/or to accurately and completely report the potential financial conflicts of interest could lead to the following: 1) Prior to publication, article rejection, or 2) Post-publication, sanctions ranging from, but not limited to, issuing a correction, reporting the inaccurate information to the authors' institution, banning authors from submitting work to ASN journals for varying lengths of time, and/or retraction of the published work.

Name: Xuelian Wei
Manuscript ID: JASN-2024-001198
Manuscript Title: LBCT: Long-term Results From an Open-label Extension Study of Atacicept for the Treatment of IgA Nephropathy
Date of Completion: September 13, 2024
Disclosure Updated Date: August 25, 2024
