# Supplementary material for: Long-Term Results from an Open-Label Extension Study of Atacicept for the Treatment of IgA Nephropathy
Source: J Am Soc Nephrol. 2024 Oct 26;36(4):679–87. doi: 10.1681/ASN.0000000541 (PMC7616790; doi:10.1681/ASN.0000000541)
Supplement: Supplementary file 2 [file jasn-36-679-s002.pdf]

Long-Term Results from an Open-Label Extension Study of

Atacicept for the Treatment of IgA Nephropathy

Supplemental Material

Table of Contents

List of Investigators 2

Supplemental Figure 1. Serum IgG, IgA, and IgM Percentage Change Through Week 96.......... 4

Supplemental Table 1. Frequency and Types of Infections.. 6

Supplemental Table 2. Key End Points in Placebo Switch Group 8

Supplemental Table 3. Summary of Participants with Missing Data for Key Variables 9

Statistical Analysis Plan

List of Investigators

Site	Principal Investigator	Location	Country
102	Umanath, Kausik	Henry Ford Hospital	United States
105	Kopyt, Nelson	Northeast Clinical Research Center	United States
106	Lafayette, Richard	Stanford University Medical Center	United States
107	Singh, Harmeet<br>Yalavarthy, Rajesh*	Western Nephrology and Metabolic Bone Disease, PC - Arvada	United States
111	Campbell, Kirk	Icahn School of Medicine at Mount Sinai	United States
112	Gohh, Reginald<br>Shah, Ankur*	Rhode Island Hospital	United States
113	Zhang, Jingjing	Thomas Jefferson University	United States
114	Kumar, Jayant	Renal Medicine Associates	United States
151	Barbour, Sean	St. Paul's Hospital	Canada
201	Bouquegneau, Antoine	Centre Hospitalier Universitaire (CHU) de Liege	Belgium
202	Maes, Bart	Algemeen Ziekenhuis Delta VZW (AZ Delta Roeselare)	Belgium
203	Speeckaert, Marijn	University Hospital Gent	Belgium
301	Vielhauer, Volker	Klinikum der Universitaet Muenchen - Campus Innenstadt	Germany
303	Hugo, Christian	Universitaetsklinikum Carl Gustav Carus Dresden	Germany
304	Nitschke, Martin	Universitaetsklinikum Schleswig-Holstein - Campus Luebeck	Germany
401	Tokgoz, Bulent	Erciyes University Faculty of Medicine	Turkey
402	Eren, Necmi	Kocaeli University Medical School - Internal Medicine - Nephrology	Turkey
453	Rydzewski, Andrzej	Panstwowy Instytut Medyczny MSWiA - Klinika Chorob Wewnetrznych, Nefrologii i Transplantologii	Poland
503	Doulton, Timothy	Kent and Canterbury Hospital	United Kingdom
504	Power, Albert	Southmead Hospital	United Kingdom
505	Hall, Matthew	Nottingham City Hospital	United Kingdom
509	Willcocks, Lisa	Cambridge University - Addenbrooke’s Hospital	United Kingdom
601	Tesar, Vladimir	Vseobecna fakultni nemocnice v Praze	Czech Republic
602	Rychlik, Ivan	Fakultni nemocnice Kralovske Vinohrady	Czech Republic
651	Goumenos, Dimitrios	University General Hospital of Patras	Greece

652	Papagianni, Aikaterini<br>Stangou, Maria*	Hippokration General Hospital of Thessaloniki	Greece
653	Boletis, Ioannis<br>Marinaki, Smaragdi*	General Hospital of Athens - Laiko	Greece
654	Stylianou, Konstantinos	University General Hospital of Heraklion	Greece
655	Zermpala, Synodi	General Hospital of Nikaia Ag. Panteleimon	Greece
656	Ntounousi, Evangelia	University General Hospital of Ioannina	Greece
704	Gang, Sishir	Muljibhai Patel Urological Hospital	India
706	Alexander, Suceena	Christian Medical College Hospital Vellore	India
707	Pandey, Rajendra	IPGMER & SSKM & R Hospital	India
712	Dalal, Sonal	Sterling Hospital	India
714	R Sunil	Kempegowda Institute of Medical Sciences and Research Centre	India
717	Khetan, Prakash	Kingsway Hospital	India
719	Jain, Alok	Apex Hospitals Pvt Ltd	India
803	Park, Hyeong Cheon	Gangnam Severance Hospital, Yonsei University Health System	South Korea
804	Kim, Dong Ki	Seoul National University Hospital	South Korea
805	Kim, Sung Gyun	Hallym University Sacred Heart Hospital	South Korea
807	Kim, Beom Seok	Severance Hospital, Yonsei University Health System	South Korea
901	Levidiotis, Vicki<br>Pedagogos, Eugenia*	Western Health	Australia
902	Francis, Ross	Princess Alexandra Hospital	Australia
903	Ryan, Jessica	Monash Health	Australia
904	Phoon, Richard	Westmead Hospital	Australia
951	Yahya, Rosnawati	Hospital Kuala Lumpur	Malaysia
952	Bin Mohd Nor, Fariz Safhan	Hospital Tengku Ampuan Afzan	Malaysia
954	Lim, Soo Kun	University Malaya Medical Centre (UMMC)	Malaysia
955	Teng, Kok Seng	Hospital Taiping	Malaysia

*Transition to new primary investigator.

Supplemental Figure 1. Serum IgG, IgA, and IgM Percentage Change Through Week 96

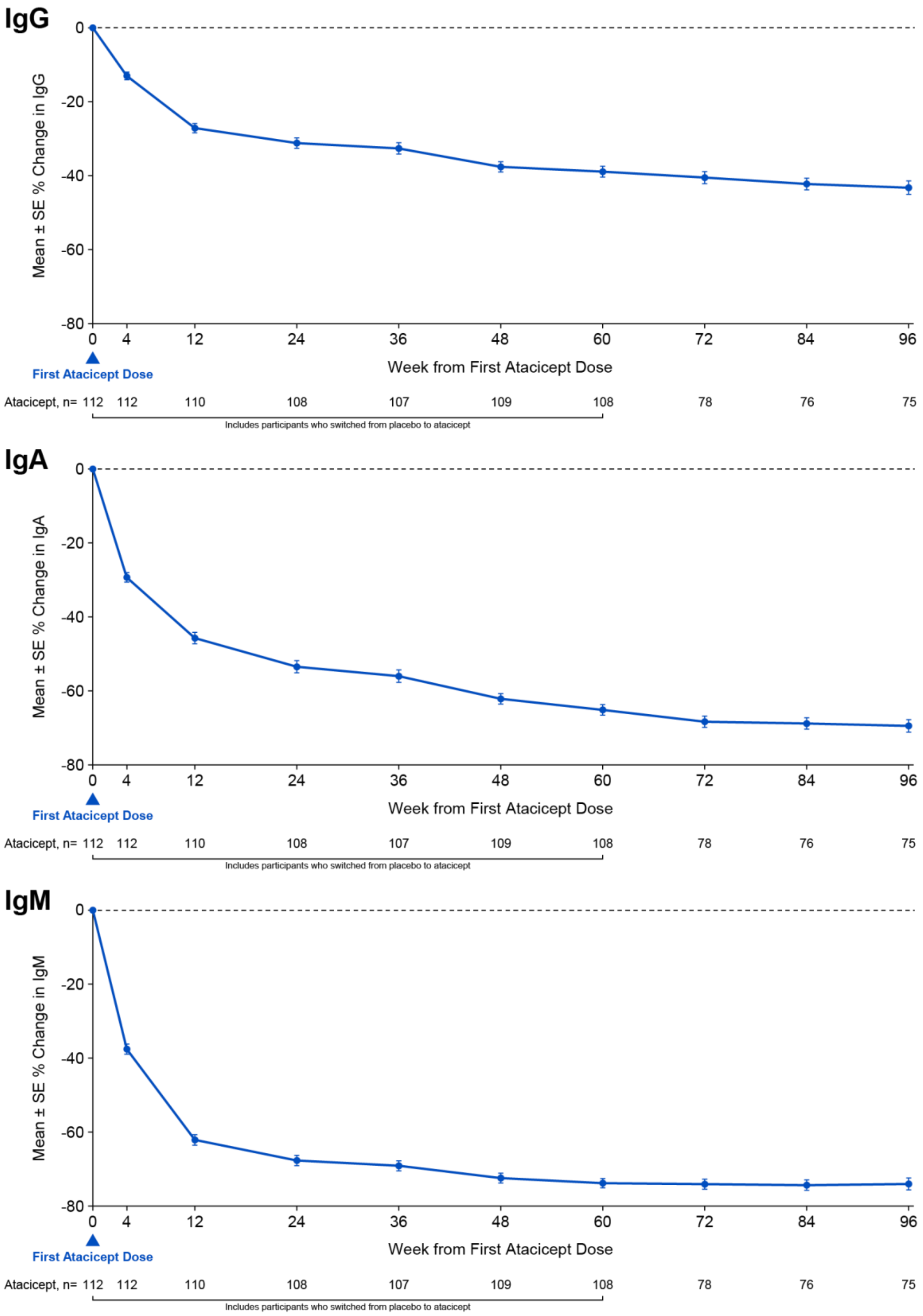

FDA=Food and Drug Administration; SE=standard error; US=United States.

Percentage changes from baseline were computed using US FDA-endorsed mixed-effects modeling.

Atacicept group includes all participants receiving any atacicept dose at any timepoint, with baseline defined as the last available measurement prior to the first dose of atacicept.

Supplemental Table 1. Frequency and Types of Infections

	**Double-Blind Baseline to Week 36**		**Open-Label Extension Week 36 to 96***
**Participants, n (%)**	**Placebo n=34**	**All Atacicept n=82**	**Atacicept 150 mg n=111**
Infections and infestations	11 (32)	35 (43)	43 (39)
Occurring in >1 participant in any group			
COVID-19	6 (18)	21 (26)	11 (10)
URTI	0	5 (6)	14 (13)
Nasopharyngitis	1 (3)	4 (5)	12 (11)
Influenza	1 (3)	1 (1)	5 (5)
Urinary tract infection	1 (3)	4 (5)	3 (3)
Viral infection	2 (6)	2 (2)	1 (1)
Bronchitis	0	1 (1)	3 (3)
Conjunctivitis	0	1 (1)	4 (4)
Gastroenteritis	0	1 (1)	2 (2)
Pneumonia	0	0	3 (3)
Tonsillitis	0	2 (2)	1 (1)
Fungal skin infection	0	0	2 (2)

Gastroenteritis viral	0	1 (1)	2 (2)

LRTI=lower respiratory tract infection; URTI=upper respiratory tract infection.

*Week 96 cut-off includes all safety data as of June 03, 2024, including visits past Week 96. Adverse events were considered treatment-emergent during the open-label extension period if they started after the first dose of open-label atacicept 150 mg through the end of the study. This group represents the 80 participants randomized to atacicept and the 31 participants randomized to placebo who entered the open-label extension.

Supplemental Table 2. Key End Points in Placebo Switch Group

Mean	Placebo<br>Study Week 0 to 36	Atacicept 150 mg<br>Study Week 36 to 96[b]
Gd-IgA1 % change ± SE	-7 ± 6<br>n=29	-54 ± 5<br>n=29
Change in % participants with hematuria[a] (95% CI)	-5 (-26, -0.1)<br>n=19	-72 (-90, -47)<br>n=18
UPCR % change ± SE	+3 ± 12<br>n=30	-49 ± 7<br>n=29
eGFR slope, mL/min/1.73m^2/year ± SE	-3.2 ± 2.4<br>n=34	-0.4 ± 0.9<br>n=30

CI=confidence interval; eGFR=estimated glomerular filtration rate; Gd-IgA1=galactose-deficient IgA1; SE=standard error; UPCR=urine protein to creatinine ratio.

a. Analysis was limited to participants with hematuria 1+ or higher at baseline.
b. Gd-IgA1, UPCR, and eGFR slope results were modeled using 31 placebo switch participants.

Supplemental Table 3. Summary of Participants with Missing Data for Key Variables

Study Week	0	4	12	24	36	48	60	72	84	96
**Gd-IgA1**										
Expected Participants, n	113	113	113	82	113	82	31	82	NA	82
Participants with Data, n	111	81	108	78	107	79	29	77	NA	74
Participants with Missing Data, n	2	32	5	4	6	3	2	5	NA	8
**Hematuria**										
Expected Participants, n	63	63	63	63	63	63	63	44	44	44
Participants with Data, n	63	63	62	60	60	61	61	43	41	40
Participants with Missing Data, n	0	0	1	3	3	2	2	1	3	4
**UPCR**										
Expected Participants, n	113	NA	113	113	113	113	113	82	82	82
Participants with Data, n	112	NA	110	107	109	109	108	78	74	75
Participants with Missing Data, n	1	NA	3	6	4	4	5	4	8	7
**eGFR**										
Expected Participants, n	113	113	113	113	113	113	113	82	82	82
Participants with Data, n	112	112	110	108	108	109	108	78	76	75
Participants with Missing Data, n	1	1	3	5	5	4	5	4	6	7

Note: The participants who were randomized to receive placebo in 36-week, randomized, double-blind phase contributed only up to 60-week follow-up data in the data analysis evaluating active atacicept treatment effect.

Missing data includes missing due to premature discontinuation, samples could not be evaluated, or participants missing visits while on study.

eGFR=estimated glomerular filtration rate; Gd-IgA1=galactose-deficient IgA1; NA=not applicable; UPCR=urine protein to creatinine ratio.

Docusign Envelope ID: E39561FE-8723-4250-B198-4822CBF9C70B

STATISTICAL ANALYSIS PLAN

**Protocol Title:**	A Phase 2b/3, Multi-part, Randomized, Double-Blinded, Placebo-Controlled Study to Evaluate the Efficacy and Safety of Atacicept in Subjects with IgA Nephropathy (IgAN)
**Protocol Number:**	VT-001-0050
**Scope of Analysis Plan**	Phase 2b (Parts A and B)
**Protocol Version/Date:**	Protocol V4.0 / 10 March 2022 (Version 1.0 SAP)<br>Protocol V6.0 / 23 August 2023 (Version 2.0 and 3.0 SAP)
**Sponsor:**	Vera Therapeutics, Inc.<br>8000 Marina Boulevard, Suite 120<br>Brisbane, CA 94005
**SAP Version/Date:**	V 1.0 / 09 December 2022<br>V 2.0 / 06 May 2024<br>V 3.0 / 11 July 2024 (**Current Version**)

CONFIDENTIAL

This document is the property of Vera Therapeutics, Inc. It is intended for restricted use only and may not in full or part be passed on, reproduced, published, or used without express permission of Vera Therapeutics, Inc.™ Copyright. All rights reserved.

Docusign Envelope ID: E39561FE-8723-4250-B198-4822CBF9C70B

SAP SIGNATURE PAGE

Protocol Title:	A Phase 2b/3, Multi-part, Randomized, Double-Blinded, Placebo-Controlled Study to Evaluate the Efficacy and Safety of Atacicept in Subjects with IgA Nephropathy (IgAN)
Protocol Number:	VT-001-0050 V6.0 / 23 August 2023
SAP Version/Date:	VT-001-0050-Phase2b-SAP-V3.0 / 11 July 2024

We, the undersigned, have reviewed and approved this Statistical Analysis Plan:

Signature **Date**

DocuSigned by:
Xuelian Wei
CA5BAF5E6FE04EB...
11-Jul-2024 | 10:39 PDT

Xuelian (Lilian) Wei
VP of Biostatistics, Vera Therapeutics, Inc

DocuSigned by:
Lijie Zhong
150206740FD44D9...
11-Jul-2024 | 11:53 PDT

Lijie Zhong
SVP of Biometrics, Vera Therapeutics, Inc.

DocuSigned by:
[illegible]
6C02CD296FF9432...
11-Jul-2024 | 11:56 PDT

Amit Sharma
EVP of Clinical Research, Vera Therapeutics, Inc.

DocuSigned by:
Robert Brenner
6BBE8610221E4BC...
11-Jul-2024 | 12:03 PDT

Robert Brenner
Chief Medical Officer, Vera Therapeutics, Inc.

Docusign Envelope ID: E39561FE-8723-4250-B198-4822CBF9C70B

TABLE OF CONTENTS

SAP SIGNATURE PAGE 2
LIST OF ABBREVIATIONS 5
1 INTRODUCTION 7
2 STUDY OVERVIEW 7
2.1 Study Objectives 7
2.1.1 Primary Efficacy Objective 7
2.1.2 Secondary Efficacy Objective 7
2.1.3 Exploratory Objectives 8
2.1.4 Safety Objective 8
2.2 Study Design 8
2.2.1 Overview 8
2.2.2 Randomization and Blinding 14
2.2.3 Study Drug 14
2.2.4 Sample Size Determination 14
2.3 Study Endpoints 14
2.3.1 Primary Efficacy Endpoint 14
2.3.2 Secondary Efficacy Endpoint 15
2.3.3 Exploratory Endpoints 15
2.3.4 Safety Endpoints 16
3 STATISTICAL METHODOLOGY 16
3.1 General Considerations 16
3.1.1 Analysis Day 16
3.1.2 Definition of Period 16
3.1.3 Definition of Baseline, First Dose, and Last Dose 17
3.1.4 Analysis Visits 18
3.1.5 Summary Statistics 22
3.1.6 Handling of Dropouts and Missing Data 23
3.2 Analysis Populations 24
3.2.1 Screening Population 24
3.2.2 Intent-to-Treat (ITT) Population 24
3.2.3 Full Analysis Set (FAS) Population 24
3.2.4 Per-Protocol (PP) Population 24
3.2.5 Safety Population 24
3.2.6 Pharmacokinetics Population 24
3.2.7 Atacicept Treated Population 24
3.3 Subject Data and Study Conduct 25
3.3.1 Subject Disposition 25
3.3.2 Protocol Deviations 25
3.3.3 Analysis Populations 26
3.3.4 Demographic and Baseline Characteristics 26
3.3.5 Medical History 27
3.3.6 Prior and Concomitant Medications 27
3.3.7 Study Drug Exposure and Compliance 27
3.4 Efficacy Assessments 29
3.4.1 Primary Efficacy Endpoint 29

Docusign Envelope ID: E39561FE-8723-4250-B198-4822CBF9C70B

3.4.2 Secondary Efficacy Endpoint 30
3.4.3 Sensitivity Analysis 31
3.4.4 Exploratory Endpoints 31
3.5 Safety Assessments 39
3.5.1 Adverse Events (AEs) 39
3.5.2 Clinical Laboratory Tests 41
3.5.3 Vital Signs 41
3.5.4 Electrocardiograms (ECG) 41
3.5.5 Physical Examinations 42
3.5.6 EuroQol (EQ-5D-5L) Study Questionnaire 42
4 INDEPENDENT DATA MONITORING COMMITTEE 42
5 MID-STUDY ANALYSIS AND FINAL ANALYSIS TIMING 43
5.1 Week 24 Primary Endpoint Analysis 43
5.2 End of Part A Analysis at Week 36 Data Cut 43
5.3 OLE Week 96 Analysis at Week 96 Data Cut 43
5.4 Final Analysis 43
6 CHANGES FROM PROTOCOL-SPECIFIED STATISTICAL ANALYSES 44
6.1 Changes from Protocol-specific Statistical Analyses in Version 1 SAP 44
6.2 Changes from Version 1 SAP 44
6.3 Changes from Version 2 SAP 45
7 PROGRAMMING SPECIFICATIONS 46

LIST OF TABLES

Table 1: Phase 2b: Schedule of Assessments 11
Table 2: Analysis Window Relative to DB Baseline for Efficacy and Lab Parameters 19
Table 3: Analysis Window Relative to OLE Baseline for Efficacy and Lab Parameters 19
Table 4: Analysis Window Relative to ATRT Baseline for Selected Efficacy Parameters 20
Table 5: Analysis Window Relative to DB Baseline for Vital Sign Parameters 21
Table 6: Analysis Window Relative to OLE Baseline for Vital Sign Parameters 22

LIST OF FIGURES

Figure 1: Study Schema 10

LIST OF APPENDICES

Appendix 2: Laboratory Tests 47
Appendix 3: SAS Code 48
Appendix 3: References 55

Docusign Envelope ID: E39561FE-8723-4250-B198-4822CBF9C70B

LIST OF ABBREVIATIONS

Abbreviation	Definition
AE	Adverse event
AESI	Adverse event of special interest
ATC	Anatomical therapeutic chemical
BMI	Body mass index
CI	Confidence interval
CKD-EPI	Chronic Kidney Disease Epidemiology Collaboration
CRF	Case report form
CRO	Contract research organization
CSR	Clinical Study Report
DB	Double-Blind
ECG	Electrocardiogram
eGFR	Estimated glomerular filtration rate
EOT	End of treatment
EQ-5D-5L	EuroQol 5-Dimension-5 Level Questionnaire
ET	Early Termination
FAS	Full analysis set
FU	Follow-up
Gd-IgA1	Galactose-deficient IgA1
HbA1c	Hemoglobin-A1c
iDMC	Independent data monitoring committee
IMP	Investigational Medicinal Product
IgA, IgG, IgM	Immunoglobin A, Immunoglobin G, Immunoglobin M
IgAN	IgA Nephropathy
ISR	Injection site reaction
ITT	Intention-to-treat
IWRS	Interactive web response system
LLOQ	Lower Limit of Quantification
LS Means	Least Squares Means
MedDRA	Medical Dictionary for Regulatory Activities
MMRM	Mixed-effects model for repeated measures
OLE	Open-Label Extension
PK	Pharmacokinetics
PP	Per-protocol
PT	Preferred term
Q1, Q3	First, Third quartile
QW	Once weekly
RAASi	Renin-Angiotensin-Aldosterone System (inhibitor)
SAE	Serious adverse event
SAP	Statistical Analysis Plan
SC	Subcutaneous
SD	Standard deviation
SGLT2i	Sodium-Glucose-Co-Transport-2 (inhibitor)
SOC	System organ class

Docusign Envelope ID: E39561FE-8723-4250-B198-4822CBF9C70B

Abbreviation	Definition
SMQ	Standardized MedDRA Query
TEAE	Treatment-emergent adverse event
TESAE	Treatment-emergent serious adverse event
ULOQ	Upper Limit of Quantification
UACR	Urine albumin to creatinine ratio
UPCR	Urine protein to creatinine ratio
VAS	Visual Analog Scale
WOCBP	Woman of childbearing potential
WHO	World Health Organization

Docusign Envelope ID: E39561FE-8723-4250-B198-4822CBF9C70B

1 INTRODUCTION

This Statistical Analysis Plan (SAP) applies to Study VT-001-0050 entitled, *A Phase 2b/3, Multi-part, Randomized, Double-Blinded, Placebo-Controlled Study to Evaluate the Efficacy and Safety of Atacicept in Subjects with IgA Nephropathy (IgAN).* This current SAP pertains to the analyses planned for the Phase 2b portion of this study. A separate SAP (or SAPs) will be developed for the Phase 3 portion of the study.

Study VT-001-0050 includes a Phase 2b portion, which is the focus of this SAP. The Phase 2b portion of the study consists of Part A, a 36-week double-blind (DB), randomized treatment period with participants randomized to atacicept 150 mg, 75 mg, or 25 mg subcutaneous (SC) once weekly (QW) vs placebo (2:2:1:2), followed by Part B, a 60-week open-label extension (OLE) during which all participants who completed the 36-week blinded treatment period on-treatment received open label atacicept 150 mg SC QW. For full details of the study design, see the study protocol.

Version 1.0 SAP was developed based on study protocol Version 4.0 (dated 10 March 2022) to support two pre-specified DB interim analyses. Version 1.0 SAP included detailed efficacy analyses for DB period, but limited analyses for OLE period. Version 1.0 of the SAP was finalized prior to the database lock for the primary endpoint analysis which occurred at the Week 24 data cut. This original SAP will be referred to as “Phase 2b Version 1 SAP” in later sections.

Version 2.0 SAP was developed based on the study Protocol Version 6.0 (dated 23 August 2023), which included detailed efficacy analyses for open-label extension data and will be referred to as “Phase 2b Version 2 SAP” in later sections.

The current SAP, Version 3.0 SAP, is developed based on the study Protocol Version 6.0 (dated 23 August 2023), which will include new efficacy analyses based on enriched sample size of atacicept treated subjects to assess long- term open-label extension data. This SAP will be referred to as “Phase 2b Version 3 SAP” in later sections.

2 STUDY OVERVIEW

2.1 Study Objectives

2.1.1 Primary Efficacy Objective

- Evaluate the effect of atacicept compared to placebo on change in proteinuria in adult subjects with immunoglobulin A (IgA) Nephropathy (IgAN) at Week 24.

2.1.2 Secondary Efficacy Objective

- Evaluate the effect of atacicept compared to placebo on change in proteinuria in adult subjects with IgAN at Week 36.

Docusign Envelope ID: E39561FE-8723-4250-B198-4822CBF9C70B

2.1.3 Exploratory Objectives

- Evaluate the effect of atacicept on change in proteinuria in adult subjects with IgAN at all post-baseline visits.
- Evaluate the effect of atacicept on change in estimated glomerular filtration rate (eGFR) at all post-baseline visits.
- Evaluate the effect of atacicept on hematuria resolution (i.e., achieving hematuria of negative or trace) at all post-baseline visits for subjects with hematuria of 1+ or above at baseline.
- Evaluate the effect of atacicept on hematuria improvement (i.e., achieving hematuria >= 1 category decrease) at all post-baseline visits for subjects with hematuria of 1+ or above at baseline.
- Evaluate the effect of atacicept on change in serum galactose-deficient IgA1 (Gd-IgA1) levels, serum immunoglobulin levels (i.e., IgA, IgG, and IgM), and complement levels (i.e., C3 and C4) at all post-baseline visits.
- Evaluate serum pharmacokinetics (PK) and atacicept anti-drug antibodies (ADA).

2.1.4 Safety Objective

- Evaluate the safety and tolerability of atacicept.

2.2 Study Design

2.2.1 Overview

This is a Phase 2b, multicenter, randomized, double-blind, placebo-controlled study to evaluate the safety and efficacy of atacicept in subjects with IgAN. Approximately 105 subjects were to be randomized 2:2:1:2 to atacicept 150 mg, atacicept 75 mg, atacicept 25 mg, or matching placebo.

- Atacicept 150 mg QW SC injections (N=30)
- Atacicept 75 mg QW SC injections (N=30)
- Atacicept 25 mg QW SC injections (N=15)
- Placebo-to-match QW SC injections (N=30)

Subjects were to be stratified by screening eGFR, calculated by the central laboratory, between subjects who have values eGFR ≥ 30 and < 45 mL/min/1.73m2 and those subjects who have an eGFR ≥ 45 mL/min/1.73m2 as per the Chronic Kidney Disease Epidemiology Collaboration (CKD-EPI) equation. Approximately 20% of the subjects were to be randomized with an eGFR between 30 and < 45 mL/min/1.73m2.

The study is composed of a (up to) 4-week Screening Period, a 36-week DB Period (Part A), followed by a 60-week OLE Period (Part B), and a 26-week Safety Follow-up (FU) Period. Upon completion of Part A, including attendance at Weeks 24 and 36 Visits for completion of all key study assessments, all subjects were to be offered open-label treatment with

Docusign Envelope ID: E39561FE-8723-4250-B198-4822CBF9C70B

atacicept 150 mg SC QW in OLE period. Subjects who discontinued study drug prior to completion of the 96-week total treatment duration were to complete an Early Termination (ET) Visit and have a 26-week follow-up period. After the last subject completed the Week 36 Visit or completed an Early Termination Visit, the study was to be unblinded. The study was conducted on an outpatient basis.

At selected sites, a subgroup of subjects (approximately 32 subjects, with 8 subjects per each Part A treatment arm) enrolled were to have additional PK sampling visits that were to be done at Day 2 and/or Day 3, approximately 24 and 48 hours after the first dose, respectively.

The target study population included male or female subjects with IgAN who had persistent proteinuria and remained at high risk of disease progression despite being on a stable prescribed regimen for at least 12 weeks with a renin-angiotensin-aldosterone system (inhibitor) (RAASi) that was at the maximum labeled or tolerated dose.

Study visits were to occur at Screening, Day 1, Weeks 2, 4, 12, 24, and 36 in DB period. Subjects were then to continue treatment in OLE period with visits at Weeks 38, 40, 48, 60, 72, 84, and 96.

There was to be a 26-week follow-up after the End-of-Treatment visit (EOT, Week 96). Subjects discontinuing study intervention early were to be required to have an Early Termination (ET) Visit within 10 days of the last dose, and safety follow up at 12 and 26 Weeks from the ET visit. Assessments to be done at each visit can be found in the Schedule of Assessments (Table 1).

The number of global study sites was to be approximately 60, with approximately 105 subjects randomized. The study schema is illustrated in the following flow chart.

Docusign Envelope ID: E39561FE-8723-4250-B198-4822CBF9C70B

Figure 1: Study Schema

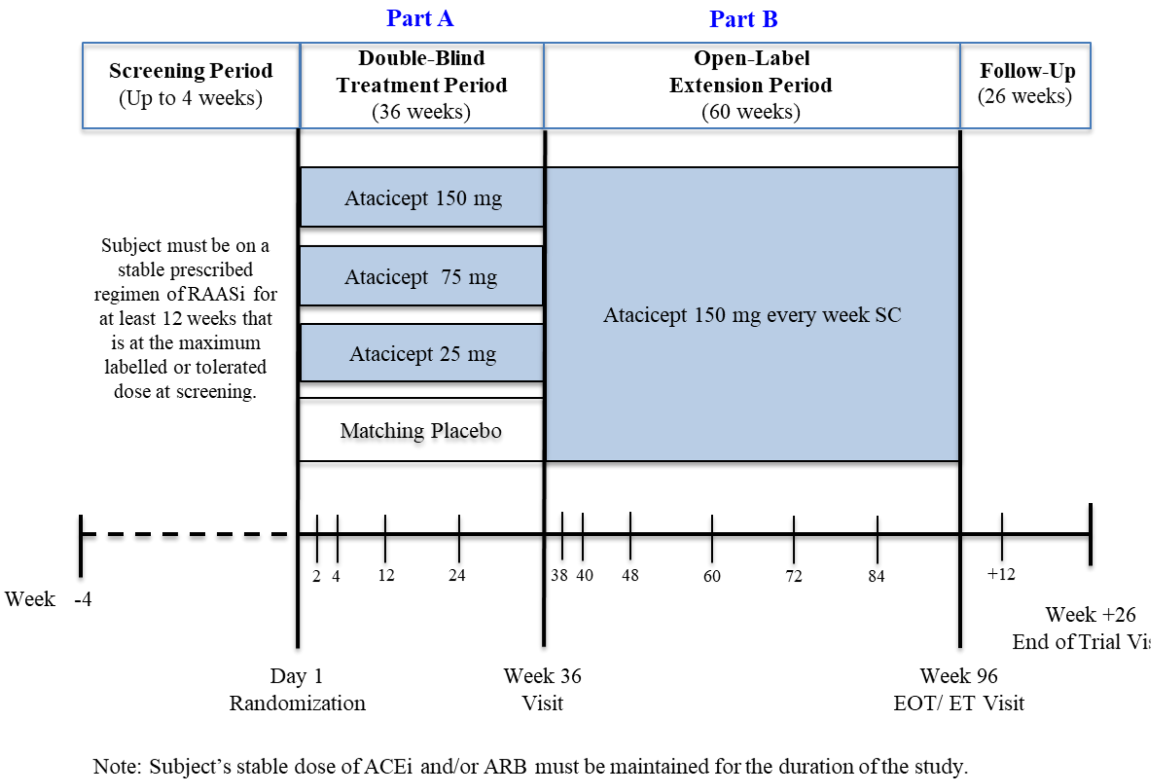

ACEi = angiotensin converting enzyme inhibitor; ARB = angiotensin II receptor blocker; EOT = end of treatment; ET = early termination; RAASi = renin-angiotensin-aldosterone system inhibitor; SC = subcutaneous.

*For Czech Republic only, additional visits were conducted at Week 1 and Week 37.

*For Germany only, additional visits were added at Week 1 and Week 3.

Docusign Envelope ID: E39561FE-8723-4250-B198-4822CBF9C70B

Table 1: Phase 2b: Schedule of Assessments

**Study Period**	**Screening**	**PART A[s] Blinded Treatment Period**						**PART B[s] Open-Label Extension Period (OLE)**							**Follow-Up**	
**Week**	**-4**	**0**	**2**	**4**	**12**	**24**	**36r**	**38**	**40**	**48**	**60**	**72**	**84**	**96/ET**	**+12**	**+26**
**Study Day**	**-28 to -1**	**1**	**15**	**29**	**85**	**169**	**253**	**267**	**281**	**337**	**421**	**505**	**589**	**673**	**From W96/ET Visit**	
**Visit Window (±day)**			**±5**	**±5**	**±10**	**±10**	**±5**	**±5**	**±5**	**±10**	**±10**	**±10**	**±10**	**±10**	**±5**	**±10**
Informed consent	X															
PK sub-study informed consent[a]	X															
Inclusion/exclusion criteria Review[b]	X	X														
Randomization		X														
Demographic data	X															
Medical history[c]	X															
Optional remote visit[d]				X				X	X		X		X			
**Assessments**																
Vital signs, weight, height[e]	X	X	X	X	X	X	X	X	X	X	X	X	X	X	X	X
12-lead ECG	X	X				X								X		
Complete physical examination	X					X								X		
Symptom-driven physical examination		X	X	X	X		X	X	X	X	X	X	X		X	X
Tuberculosis assessment	X															
Study questionnaire (EQ-5D-5L)		X				X				X		X		X		
**Laboratory Assessments**																
Routine hematology, chemistry, urinalysis[f]	X	X	X	X	X	X	X	X	X	X	X	X	X	X	X	X
TSH	X															
Serum Virology[g]	X															
Serum pregnancy test[h]	X															
HbA1c	X															
Urine pregnancy test[i]		X		X	X	X	X	X	X	X	X	X	X	X	X	X

**Study Period**	**Screening**	**PART A[s] Blinded Treatment Period**						**PART B[s] Open-Label Extension Period (OLE)**							**Follow-Up**	
**Week**	**-4**	**0**	**2**	**4**	**12**	**24**	**36r**	**38**	**40**	**48**	**60**	**72**	**84**	**96/ET**	**+12**	**+26**
**Study Day**	**-28 to -1**	**1**	**15**	**29**	**85**	**169**	**253**	**267**	**281**	**337**	**421**	**505**	**589**	**673**	**From W96/ET Visit**	
**Visit Window (±day)**			**±5**	**±5**	**±10**	**±10**	**±5**	**±5**	**±5**	**±10**	**±10**	**±10**	**±10**	**±10**	**±5**	**±10**
24-hour urine collection for quantitative analysis[j]	X	X			X	X	X			X	X	X	X	X		
Archival kidney biopsy/ Pre-treatment kidney biopsy[k]	X															
**Pharmacokinetic Assessments**																
Serum atacicept[l]		X	X	X	X	X	X			X		X		X	X	X
Anti-drug antibodies		X				X	X			X		X		X	X	X
**Pharmacodynamic Assessment**																
Immunoglobulins[m] (IgG,IgA,IgM)	X[l]	X		X	X	X	X		X	X	X	X	X	X	X	X
Complement (C3, C4)		X				X	X							X	X	X
Gd-IgA1		X		X	X	X	X			X		X		X	X	X
Serum BLyS & APRIL level[n]		X				X	X									
Exploratory Biomarkers[o]		X		X	X	X	X		X	X	X		X	X	X	X
**Safety and Study Drug**																
Prior/Concomitant medications and procedures	Continuous															
Adverse events	Continuous															
Local injection tolerability		Continuous														
IMP accountability			X	X	X	X	X	X	X	X	X	X	X	X		
IMP administration[p]		X[p]	X	X	X	X	X	X	X	X	X	X	X			
IMP dispensing to subject		X		X	X	X	X		X	X	X	X	X			
Pregnancy test dispensing to subject[q]				X	X	X	X		X	X	X	X	X			

CKD-EPI=Chronic Kidney Disease Epidemiology Collaboration; ECG=electrocardiogram; eGFR=estimated glomerular filtration rate; EQ-5D-5L=EuroQol 5-Dimension-5 Level Questionnaire; ET=early termination; Gd-IgA1=galactose deficient IgA1; HbA1c=hemoglobin-A1c; Ig=immunoglobulin; IgAN=IgA Nephropathy; IMP=investigational medicinal product; ISR=injection site reaction; PGx=pharmacogenetics; PK=pharmacokinetics; TSH=Thyroid stimulating hormone; WOCBP=woman of childbearing potential.

Docusign Envelope ID: E39561FE-8723-4250-B198-4822CBF9C70B

a. PK Sub-study: Additional PK sampling will be done on Days 2 and/or 3 in a subgroup of study subjects (approximately 8 subjects per treatment group).
b. Subject eligibility (based on screening assessments of the inclusion and exclusion criteria) must be checked again on Day 1 prior to randomization. Predose assessments on Day 1 are considered baseline measurements prior to the first dose of study drug.
c. IgAN clinical diagnosis, medical history, medications, previous vaccinations, surgery/procedures. Documentation of IgAN on biopsy within 10 years. See Protocol Section 4.
d. At the investigator's discretion, the study visit(s) can be conducted in-clinic or remotely. If conducted remotely, the visit will include alternative methods for safety, efficacy, and distribution/collection of Investigational Medicinal Product (IMP), including but not limited to phone/video contact, alternative location for biologic sample collection, alternative secure delivery of IMP and home health care (if available).
e. Vital signs measured per Protocol Section 6.6.2. Order of procedures done per Protocol Section 6. Height to be measured at screening only.
f. eGFR will be calculated using the CKD-EPI formula. Routine urinalysis: A sample from "fresh" urine will be collected during the scheduled clinic visit. Subjects will be provided with a urine cup and instructions for a mid-stream collection to provide a clean-catch specimen. For Optional Remote Visits, urine should be collected via clean catch.
g. Includes testing for Hepatitis B, Hepatitis C and HIV. Testing for HBV will occur monthly ±10 days.
h. Women of childbearing potential (WOCBP) only. The screening test is serum.
i. Women of childbearing potential (WOCBP) only Post screening, positive urine pregnancy test results will be confirmed by serum pregnancy testing.
j. 24-hour urine collection should begin 1 day before the study visit and should start with discarding the first morning void, and collecting all urine produced thereafter (through the day and night) up to 24 hours later (including the first morning void of the second morning). Subjects will be provided with adequate urine collection cups in advance of 24-hour urine collection timepoints.
k. A confirmed biopsy report (with 10 years of Screening Visit) is required to confirm IgAN diagnosis. If unavailable, a pre-treatment kidney biopsy is required to confirm eligibility.
l. On Day 1 only, following monitoring of ISR, a blood sample will be collected between 2-4 hours after the first study dose. After Day 1 at PK timepoints, a blood sample for PK analysis will be collected to assess levels of serum atacicept. Time and date of last dose of study drug will be collected. A separate PK sub-study will be conducted for a subgroup of subjects where additional blood samples (PK analysis) will be collected on Day 2 and/or Day 3 post-first dose of IMP
m. At Screening, IgG only.
n. Test performed if an assay is available.
o. Blood samples will be collected and stored for future clinical laboratory testing, in order to provide additional clinical data. No genetic testing will be performed on samples.
p. See Protocol Section 5.4. IMP to be administered once weekly. Injections at Randomization and Week 36 are given in the clinic. At the Week 36 Visit after completion of all study visit assessments, subjects will switch from double-blind treatment to open-label treatment.
q. Sites will provide WOCBP with enough tests to conduct pregnancy testing once per month until the next scheduled study visit during Parts A and B and will contact these subjects monthly to confirm that the pregnancy test has been performed and the results are negative.
r. At the Week 36 Visit after completion of all study visit assessments, subjects will switch from double-blind treatment to open-label treatment.
s. For Czech Republic additional visits were added at Week 1 and Week 37 and subjects will remain on site for 2 hours and monitored for hypersensitivity reactions. For Germany additional visits were added at Week 1 and Week 3.

Docusign Envelope ID: E39561FE-8723-4250-B198-4822CBF9C70B

2.2.2 Randomization and Blinding

Subjects were randomly assigned based on a predefined randomization code via the interactive web response system (IWRS) at the Day 1/ Randomization visit. The site staff, Sponsor, and contract research organization (CRO) who were directly involved in the management of the study were blinded to the subject's treatment allocation, with the exception of the selected study drug supply, and serious adverse event (SAE) reporting contact personnel. A statistical team, independent from the study management team, was to be unblinded at the time of analysis of the primary endpoint. For emergency unblinding only, corresponding treatment assignments were to be made available to the Investigator through the IWRS.

2.2.3 Study Drug

In a blinded fashion, 150 mg atacicept, 75 mg atacicept, 25 mg atacicept or matching placebo was given as QW SC injections for 36 weeks by prefilled syringe. For the OLE, open-label 150 mg atacicept was to be given as QW SC injections for 60 weeks by prefilled syringe.

2.2.4 Sample Size Determination

Sample size and power was estimated for the primary efficacy endpoint, change from baseline to Week 24 in natural log transformed urine protein to creatinine ratio (UPCR). It is assumed that the absolute difference (placebo – active) in the mean ratio of Week 24 UPCR to baseline UPCR was 0.28. The geometric mean ratio of Week 24 UPCR to baseline for placebo was expected to be 0.88. The coefficient of variation was estimated as 0.594 (i.e., pooled log standard deviation [SD] of 0.55). The expected placebo response and study intervention effect size were based on the analysis of the Phase 2 study for atacicept and placebo in IgAN and placebo responses seen in other large IgAN studies. Based on these assumptions, 25 subjects in the placebo group and a combined total of 50 subjects in the atacicept 75 mg and 150 mg groups were considered sufficient to provide at least 80% power to detect a ratio of 1.47 when the ratio under the null hypothesis is 1 [PASS 2008: two-sided two Sample t-test for ratios, α=0.05, coefficient of variation on the original scale was 0.594]. Adjusting for a 15% dropout rate, 30 subjects were to be enrolled in each of the placebo, atacicept 75 mg, and atacicept 150 mg groups to ensure 25 subjects complete Week 24 in each of these groups. The sample size of 15 for the atacicept 25 mg group was chosen to provide preliminary safety and efficacy information and was not based on statistical considerations.

2.3 Study Endpoints

2.3.1 Primary Efficacy Endpoint

The primary efficacy endpoint is the change from baseline at Week 24 in natural log transformed UPCR based on 24-hour urine sample.

Docusign Envelope ID: E39561FE-8723-4250-B198-4822CBF9C70B

2.3.2 Secondary Efficacy Endpoint

The secondary efficacy endpoint is the change from baseline at Week 36 in natural log transformed UPCR based on 24-hour urine sample.

2.3.3 Exploratory Endpoints

- Change from baseline by visit in natural log transformed UPCR based on 24-hour urine sample.
- Change from baseline by visit in natural log transformed eGFR.
- Change from baseline by visit in eGFR (using the original scale).
- Annualized rate of change in eGFR through Week 96.
- Proportion of subjects with hematuria resolution (i.e., achieving hematuria of negative or trace) by visit for subjects with hematuria of 1+ or above at baseline.
- Proportion of subjects with hematuria improvement (i.e., achieving hematuria ≥ 1 category decrease) by visit for subjects with hematuria of 1+ or above at baseline.
- Change from baseline by visit in natural log transformed Gd-IgA1, IgA, IgG, and IgM levels.
- Change from baseline by visit in natural log transformed Urine Albumin to Creatinine Ratio (UACR) based on 24-hour urine sample.
- Absolute, change from baseline, and percentage change from baseline by visit for UPCR, eGFR, Gd-IgA1, IgA, IgG, IgM, C3 and C4 levels.
- Serum concentration of atacicept at pre-specified time points.
- Anti-drug antibody assessment at pre-specified time points.

<u>Key Changes in the OLE Week 96 and Final Analyses:</u>

After the End of Part A analysis, the Sponsor decided to conduct an OLE Week 96 analysis to evaluate the long-term eGFR effect to inform the sample size calculation for the Phase 3 part of the study.

The following changes for endpoints will be made in the OLE Week 96 analysis and the Final analysis:

- The exploratory endpoints of “change from baseline by visit in natural log transformed C3 and C4 levels” will be removed, since C3 and C4 are not closely tied to Atacicept’s Mechanism of Action (MOA) in IgAN.
- The following exploratory endpoints will be added based on new clinical evidence for IgAN treatment.
  - Change from baseline by visit in eGFR (using the original scale).
  - Annualized rate of change in eGFR through Week 96.
  - Proportion of subjects with hematuria resolution (i.e., achieving hematuria of negative or trace) by visit for subjects with hematuria of 1+ or above at baseline.
  - Proportion of subjects with hematuria improvement (i.e., achieving hematuria ≥ 1 category decrease) by visit for subjects with hematuria of 1+ or above at baseline.

Docusign Envelope ID: E39561FE-8723-4250-B198-4822CBF9C70B

2.3.4 Safety Endpoints

Safety endpoints include evaluation of routine clinical and laboratory tests and adverse events during the entire study.

3 STATISTICAL METHODOLOGY

There were three analyses planned in the protocol for this study: (1) Primary Endpoint Analysis at Week 24, (2) End of Part A analysis at Week 36, and (3) Final analysis.

After the End of Part A analysis, the Sponsor decided to conduct an OLE Week 96 analysis to evaluate the long-term eGFR effect to inform the sample size calculation for Phase 3 part of the study.

This SAP describes the methodologies and data presentation for the OLE Week 96 analysis and the final analysis.

3.1 General Considerations

3.1.1 Analysis Day

Analysis day will be calculated from the date of the first dose of study drug. The day of the first dose of study drug will be Day 1, and the day immediately before Day 1 will be Day -1. There will be no Day 0.

3.1.2 Definition of Period

Analysis for the DB period will include data collected during the screening and double-blinded periods: (1) all available data for subjects who prematurely discontinued the blinded study drug and did not receive any dose of OL atacicept in the OLE period; (2) all available data prior to the first dose data/time of OL atacicept for subjects who completed the double-blinded drugs and received at least 1 dose of OLE atacicept.

Analysis for the OLE period will include OLE baseline and all data collected on or after the first dose date/time of OL atacicept through the end of the study.

Analysis for the entire study period will include all the data collected throughout the study, i.e. from screening period to the end of follow-up period.

Analysis for the atacicept treatment (ATRT) period will include ATRT baseline (defined in Section 3.1.3) and data collected on or after the first dose date/time of either DB or OL atacicept (i.e., the first dose of DB atacicept for subjects originally randomized to active atacicept groups or the first dose of OL atacicept for subjects originally randomized to placebo group). For subjects who were originally randomized to the placebo and did not receive any dose of OL atacicept (eg., due to prematurely discontinued study drug or study), will be excluded from the analysis for the atacicept treatment period.

Docusign Envelope ID: E39561FE-8723-4250-B198-4822CBF9C70B

3.1.3 Definition of Baseline, First Dose, and Last Dose

Baseline for the DB period (labeled as DB baseline for future reference) is defined as the last available measurement prior to the first dose of DB study drug (i.e., it is generally the predose assessment on Day 1), except for UPCR, UACR, and other laboratory tests from 24-hour urine samples, where the geometric mean of all measurements during the screening period and prior to the first dose of study drug will be used as baseline. DB baseline is the default baseline and will be used interchangeably with baseline. The change from baseline analyses refers to the change from DB baseline analyses, which will include data from both Parts A, B, and the follow-up period (refer to Table 2 for analysis window definition).

The baseline for the OLE period (OLE baseline) is defined as the last available measurement prior to the first dose of open-label atacicept at Week 36 visit. OLE baseline will be defined for all efficacy endpoints, including UPCR, eGFR, Gd-IgA1, IgG, IgA, IgM, C3, C4, etc. OLE baseline will also be defined for all safety laboratory parameters in the OLE period. Analyses related to change from OLE baseline will be presented for the OLE Week 96 and the Final Analyses (refer to Table 3 for analysis window definition).

The baseline for the Atacicept treatment period (ATRT baseline) is defined as DB baseline for subjects originally randomized to active atacicept groups and as OLE baseline for subjects originally randomized to placebo group. Analyses related to change from ATRT baseline will be presented for the OLE Week 96 and the Final Analyses (refer to Table 4 for analysis window definition). ATRT baseline will be defined for selected efficacy endpoints, including UPCR, UACR, eGFR, Gd-IgA1, IgG, IgA, IgM, and hematuria.

The first dose date for both the DB period and entire study is the first injection date of any study drug. For subjects who completed the Week 36 visit, the last dose date for the DB period is the last injection date prior to the actual Week 36 visit date. The first dose date for the OLE period is the first injection date on or after the actual Week 36 visit date. The last dose date for the OLE period is the last injection date for the OLE Week 96 and Final analyses.

For subjects with missing Week 36 visits due to premature discontinuation of study drug, the last dose date for the DB period will be the last injection date, which should be the same as the last dose date in the “End of treatment” eCRF. The first dose and last dose dates for OLE period will be missing for such subjects.

The last dose date for the entire study will be the last dose date during the OLE period if not missing, otherwise the last dose date for the entire study will be the last date during the DB period.

The first dose date for ATRT period is the first dose date of DB period for subjects originally randomized to active atacicept groups and the first dose date of OLE period for subjects originally randomized to placebo group and took OL atacicept. The last dose date for ATRT period is the last dose date for the entire study for subjects originally randomized to active atacicept groups and the last dose date of OLE period for subjects originally randomized to placebo group.

Docusign Envelope ID: E39561FE-8723-4250-B198-4822CBF9C70B

3.1.4 Analysis Visits

All efficacy and/or lab assessments, including those from unscheduled visits and regardless of visit label recorded on the case report form (CRF), will be allocated to analysis visits included in Table 2 and Table 3. Vital sign assessments will be allocated to analysis visits included in Table 4 and Table 5. If more than one measurement is recorded within a visit window:

- If there are multiple measurements collected on the same day (even if some measurements are collected as unscheduled measurements), the arithmetic or geometric mean of all measurements will be used for that day. The visit will be treated as a scheduled visit if at least one measurement is collected as a scheduled measurement. Otherwise, this visit will be treated as an unscheduled visit.
- If a scheduled visit falls within the visit window, the measurement taken at that scheduled visit will be used. If multiple scheduled visits are available within the visit window, the measurement taken at the scheduled visit closest to the target day will be used. If there are multiple scheduled visits with equidistant from the target day, the latest one will be used.
- If no scheduled visit falls within the visit window, the measurement from the unscheduled visit closest to the target day within the window will be used. If there are multiple unscheduled measurements with equidistant from the target day, the latest one will be used.

Docusign Envelope ID: E39561FE-8723-4250-B198-4822CBF9C70B

Table 2: Analysis Window Relative to DB Baseline for Efficacy and Lab Parameters

Analysis Visit	Target Analysis Day	Earliest Analysis Day	Latest Analysis Day
Screening	-1		-1
DB Baseline (Day 1)	1		1
DB Week 2	15	2	21
DB Week 4	29	22	56
DB Week 12	85	57	126
DB Week 24	169	127	210
DB Week 36	253	211	294 for subjects not participating in OLE; the OLE first dose date/time for subjects continuing in OLE.
OLE Week 38	267	OLE first dose date/time	273
OLE Week 40	281	274	308
OLE Week 48	337	309	378
OLE Week 60	421	379	462
OLE Week 72	505	463	546
OLE Week 84	589	547	630
OLE Week 96	673	631	Week 96 visit date+30
Follow-up Week +12	From W96/ET visit +85	From W96/ET visit +31	From W96/ET visit +133
Follow-up Week +26	From W96/ET visit +183	From W96/ET visit +134	NA

Note: Analysis Day = Event Date – First Date of Dosing in DB period +1 for events on Day 1 and after. Analysis Day = Event Date – First Date of Dosing for events prior to first date of dosing in DB period. ET = early termination.

Table 3: Analysis Window Relative to OLE Baseline for Efficacy and Lab Parameters

Analysis Visit	Target Analysis Day	Earliest Analysis Day	Latest Analysis Day
OLE Baseline (Week 36)	1	1	1
OLE Week 38	15	2	21
OLE Week 40	29	22	56
OLE Week 48	85	57	126
OLE Week 60	169	127	210
OLE Week 72	253	211	294
OLE Week 84	337	295	378
OLE Week 96	421	379	Week 96 Visit Date +30
Follow-up Week +12	From W96/ET visit +85	From W96/ET visit +31	From W96/ET visit +133
Follow-up Week +26	From W96/ET visit +183	From W96/ET visit +134	NA

Note: Analysis Date = Event Date - First date of Dosing in OLE period + 1

Docusign Envelope ID: E39561FE-8723-4250-B198-4822CBF9C70B

Table 4: Analysis Window Relative to ATRT Baseline for Selected Efficacy Parameters

For Subjects Randomized to Active Atacicept Groups:

Analysis Visit	Target Analysis Day	Earliest Analysis Day	Latest Analysis Day
Screening	-1		-1
ATRT Baseline (Day 1)	1		1
Week 2	15	2	21
Week 4	29	22	56
Week 12	85	57	126
Week 24	169	127	210
Week 36	253	211	294 for subjects not participating in OLE; the OLE first dose date/time for subjects continuing in OLE.
Week 38	267	OLE first dose date/time	273
Week 40	281	274	308
Week 48	337	309	378
Week 60	421	379	462
Week 72	505	463	546
Week 84	589	547	630
Week 96	673	631	Week 96 visit date+30

Note: Analysis Day = Event Date – First Date of Dosing in DB period +1 for events on Day 1 and after.
Analysis Day = Event Date – First Date of Dosing for events prior to first date of dosing in DB period.
ET = early termination.

For Subjects Randomized to Placebo Group:

Analysis Visit	Target Analysis Day	Earliest Analysis Day	Latest Analysis Day
ATRT Baseline	1	1	1
Week 2	15	2	21
Week 4	29	22	56
Week 12	85	57	126
Week 24	169	127	210
Week 36	253	211	294
Week 48	337	295	378
Week 60	421	379	Week 96 Visit Date +30

Note: Analysis Date = Event Date - First Date of Dosing in OLE period + 1

Docusign Envelope ID: E39561FE-8723-4250-B198-4822CBF9C70B

Table 5: Analysis Window Relative to DB Baseline for Vital Sign Parameters

Analysis Visit	Target Analysis Day	Earliest Analysis Day	Latest Analysis Day	Nominal Timepoint
Screening	-1		-1	
DB Baseline (Day 1, predose)	1		The DB first dose date/time	
DB Day 1, postdose	1	DB first dose date/time	1	5 min postdose
DB Day 1, postdose	1	DB first dose date/time	1	15 min postdose
DB Day 1, postdose	1	DB first dose date/time	1	30 min postdose
DB Day 1, postdose	1	DB first dose date/time	1	1 Hour postdose
DB Day 1, postdose	1	DB first dose date/time	1	1.5 hr postdose
DB Day 1, postdose	1	DB first dose date/time	1	2 hr postdose
DB Week 2	15	2	21	
DB Week 4	29	22	56	
DB Week 12	85	57	126	
DB Week 24	169	127	210	
Week 36, pre-OLE-dose	253	211	294 for subjects not participating in OLE; the OLE first dose date/time for subjects continuing in OLE.	
Week 36, post-OLE-dose	OLE first dose date	OLE first dose date/time	OLE first dose date	5 min postdose
Week 36, post-OLE-dose	OLE first dose date	OLE first dose date/time	OLE first dose date	15 min postdose
Week 36, post-OLE-dose	OLE first dose date	OLE first dose date/time	OLE first dose date	30 min postdose
Week 36, post-OLE-dose	OLE first dose date	OLE first dose date/time	OLE first dose date	1 Hour postdose
Week 36, post-OLE-dose	OLE first dose date	OLE first dose date/time	OLE first dose date	1.5 hr postdose
Week 36, post-OLE-dose	OLE first dose date	OLE first dose date/time	OLE first dose date	2 hr postdose
OLE Week 38	267	The OLE first dose date + 1	273	
OLE Week 40	281	274	308	
OLE Week 48	337	309	378	
OLE Week 60	421	379	462	
OLE Week 72	505	463	546	
OLE Week 84	589	547	630	
OLE Week 96	673	631	Week 96 visit date +30	
Follow-up Week +12	From W96/ET visit +85	From W96/ET visit +31	From W96/ET visit +133	
Follow-up Week +26	From W96/ET visit +183	From W96/ET visit +134	NA	

Note: Analysis Day = Event Date – First Date of Dosing in DB period +1 for events on Day 1 and after. Analysis Day = Event Date – First Date of Dosing for events prior to first date of dosing in DB period.
Per protocol, Week 36 Pre-Dose records should be measured prior to the first dose of OLE, while Week 36 Post-Dose should be measured after the first dose of OLE.

Table 6: Analysis Window Relative to OLE Baseline for Vital Sign Parameters

Analysis Visit	Target Analysis Day	Low Analysis Day	High Analysis Day	Nominal Timepoint
OLE Baseline (Week 36, pre-OLE-dose)	1	1	On or prior to the OLE first dose date/time	Predose
Week 36, post-OLE-dose	1	OLE first dose date/time	OLE first dose date	5 min postdose
Week 36, post-OLE-dose	1	OLE first dose date/time	OLE first dose date	15 min postdose
Week 36, post-OLE-dose	1	OLE first dose date/time	OLE first dose date	30 min postdose
Week 36, post-OLE-dose	1	OLE first dose date/time	OLE first dose date	1 hr postdose
Week 36, post-OLE-dose	1	OLE first dose date/time	OLE first dose date	1.5 hr postdose
Week 36, post-OLE-dose	1	OLE first dose date/time	OLE first dose date	2 hr postdose
OLE Week 38	15	OLE first dose date + 1	21	
OLE Week 40	29	22	56	
OLE Week 48	85	57	126	
OLE Week 60	169	127	210	
OLE Week 72	253	211	294	
OLE Week 84	337	295	378	
OLE Week 96	421	379	Week 96 visit date +30	
Follow-up Week +12	From Week 96/ET visit +85	From Week 96/ET visit +31	From Week 96/ET visit +133	
Follow-up Week +26	From Week 96/ET visit +183	From Week 96/ET visit +134	NA	

Note: Analysis Date = Event Date - First date of Dosing in OLE period + 1.

Per protocol, Week 36 Pre-Dose records should be measured prior to the first dose of OLE, while Week 36 Post-Dose should be measured after the first dose of OLE.

3.1.5 Summary Statistics

All endpoints will be summarized using descriptive statistics. Continuous variables will be summarized descriptively using the number of observations, mean, SD, median, lower quartile (Q1), upper quartile (Q3), minimum, and maximum. Categorical variables will be summarized using frequency counts and percentages. The denominator for the percentages will be the total number of subjects in the treatment group and analysis population being presented, unless otherwise specified. All available data will be presented in the data listings by randomized treatment group and subject number.

Docusign Envelope ID: E39561FE-8723-4250-B198-4822CBF9C70B

3.1.6 Handling of Dropouts and Missing Data

For continuous endpoints such as the primary and secondary efficacy endpoints to be analyzed using the mixed-effects model for repeated measures (MMRM), no explicit imputation of missing data is needed as the MMRM analysis will be performed on observed cases and implicitly imputes missing data.

To assess the robustness of MMRM modeling results, i.e., the impact of missing data on the result from the MMRM models, a tipping point analysis will be performed as described in Section 3.4.3.2.

Date of birth of each subject is collected as birth year and month in the CRF. For the calculation of age at baseline, the day of birth will be imputed to the 15th day of the birth month.

For prior and concomitant medications, if a medication has incomplete start or stop dates, dates will be imputed to determine whether a medication should be considered prior or concomitant. If a medication start date is incomplete, the first day of the month will be imputed for missing day and January will be imputed for missing month. If a medication stop date is incomplete, the last day of the month will be imputed for missing day and December will be imputed for missing month. Incomplete start and stop dates will be listed as collected in the listing.

For continuous values of critical labs that are not able to be determined due to being less than the lower limit of quantification (LLOQ) or higher than the upper limit of quantification (ULOQ), the values will be assigned to one unit lower than the LLOQ or one unit higher than the ULOQ for any descriptive summarization or analyses performed. For example, values reported as <50 and <5.0 will be imputed to 49 and 4.9, respectively. An exception to this is any value reported as <1 or <0.1 will be imputed as 0.9 and 0.09, respectively. Similarly, values reported as >x will be imputed using the same approach. For data reported in the form of “<=x” or “>=x” where x is considered the LLOQ or ULOQ, the LLOQ or ULOQ values will be used for imputation. Original values will be displayed in the data listings.

PK concentration values that are below the limit of quantitation (BLQ) will be presented as “BLQ” in the concentration data listing. Values that are BLQ will be treated as 0 prior to the first measurable concentration, and one-half the value of the LLOQ at postdose time points.

Descriptive summaries of efficacy and safety measures will be based on observed data. No imputation of missing data will be implemented.

If it is not possible to determine whether an AE is or is not treatment emergent, or whether an AE starts in the on-treatment period, due to completely or partially missing dates, the dates will be imputed in a conservative way so that the AE will be considered as treatment emergent, i.e., starting in the treatment period.

Docusign Envelope ID: E39561FE-8723-4250-B198-4822CBF9C70B

3.2 Analysis Populations

3.2.1 Screening Population

The Screening Population consists of all subjects who sign informed consent.

3.2.2 Intention-to-Treat (ITT) Population

The ITT Population consists of all randomized subjects. Subjects will be analyzed according to randomized treatment.

3.2.3 Full Analysis Set (FAS) Population

The FAS Population consists of all randomized subjects who have received at least 1 dose of study drug. Subjects will be analyzed according to randomized treatment. Unless otherwise indicated, the FAS Population is the primary analysis population for efficacy endpoints.

3.2.4 Per-Protocol (PP) Population

The Week 24 and 36 PP populations consist of all subjects in the FAS Population who do not have any clinically predefined specific protocol deviations.

Two PP Populations (i.e., Week 24 PP and Week 36 PP) were determined through blinded review prior to Week 24 and Week 36 database locks, respectively. The PP Population will not be defined for OLE analysis and final analysis.

3.2.5 Safety Population

The Safety Population consists of all randomized subjects who receive at least 1 dose of study drug. The Safety Population is the analysis population for the safety endpoints. Subjects will be analyzed according to treatment received during the DB period. Unless otherwise indicated, the Safety Population is the primary analysis population for safety endpoints.

3.2.6 Pharmacokinetics Population

The PK population consists of all randomized subjects who were administered at least 1 dose of study drug and have at least 1 PK concentration measurement. All PK evaluations will be based on this analysis set. Subjects will be analyzed according to the treatment received during the DB period.

3.2.7 Atacicept Treated Population

The Atacicept Treated population consists of all FAS population who were administered at least 1 dose of either DB atacicept or OL atacicept. In other words, subjects who were originally randomized to placebo group and did not receive any dose of OL atacicept (eg.,

Docusign Envelope ID: E39561FE-8723-4250-B198-4822CBF9C70B

due to prematurely discontinued study drug or study) will be excluded from Atacicept Treated Population. Subjects will be pooled into 1 group, labelled as "Atacicept-Treated" group to support efficacy analyses. Subjects originally randomized to active atacicept groups will receive atacicept for up to 96 weeks, while subjects originally randomized to placebo group will receive atacicept for up to 60 weeks.

3.3 Subject Data and Study Conduct

3.3.1 Subject Disposition

The number and percentage of subjects in each of the following disposition categories as recorded on the CRF will be presented for Screening Population:

- Subjects who failed screening
- Subjects who were randomized

Subjects who failed the screen will be tabulated by the screen failure reason. The number and percentage of subjects who failed the screen due to COVID-19 will also be presented.

The number and percentage of subjects in each of the following disposition categories as recorded on the CRF will be presented by treatment and in total for the ITT Population:

- Subjects who prematurely discontinued study drug in the DB period by reason
- Subjects who completed study drug in the DB period
- Subjects who completed study drug in the DB period and continued to OLE period
- Subjects who prematurely discontinued study drug in the OLE period by reason
- Subjects who completed study drug in the OLE period (Week 96)
- Subjects who completed study drug in the OLE period and continued to safety follow-up period
- Subjects who prematurely discontinued the study by reason
- Subjects who completed the study

The primary reason for premature discontinuation from the treatment and premature discontinuation from the study will be tabulated. The number and percentage of subjects who prematurely discontinued due to COVID-19 will also be presented.

3.3.2 Protocol Deviations

Counts and percentages of subjects with CSR reportable protocol deviations by deviation category will be summarized by treatment and in total using the ITT Population. CSR reportable protocol deviations are deviations related to inclusion/exclusion criteria, conduct of the trial, subject management or subject assessment that impact the safety of the subjects or jeopardize the quality of study data. CSR reportable protocol deviation categories are described in the protocol deviation plan.

All protocol deviations (CSR reportable and CSR non-reportable) will be listed by deviation categories.

Docusign Envelope ID: E39561FE-8723-4250-B198-4822CBF9C70B

3.3.3 Analysis Populations

Counts and percentages of subjects in each analysis population will be summarized by treatment and in total using the ITT Population.

3.3.4 Demographic and Baseline Characteristics

Demographic and baseline characteristics include, but are not limited to:

- Age at baseline (years)
- Age at baseline Category 1 (<40 years, ≥40 years)
- Age at baseline Category 2: (< 45 years, ≥45 and <65 years, and ≥65 years)
- Sex
- Childbearing potential (female only)
- Race
- Ethnicity
- Country
- Region Category 1 (Asia, Europe, North America, and South America)
- Region Category 2 (Asia and Non-Asia)
- Baseline height (cm)
- Baseline weight (kg)
- Baseline body mass index (BMI) (kg/m2)
- Screening eGFR (mL/min/1.73m2)
- Screening eGFR Category 1 (eGFR<30 mL/min/1.73m2, eGFR ≥30 and <45 mL/min/1.73 m2, eGFR ≥45 mL/min/1.73 m2)
- Screening eGFR Category 2 (eGFR<15 mL/min/1.73m2, eGFR ≥15 and <30 mL/min/1.73 m2, eGFR ≥30 and <60 mL/min/1.73 m2, eGFR ≥60 and <90 mL/min/1.73 m2, eGFR ≥90 mL/min/1.73 m2)
- Baseline eGFR (mL/min/1.73 m2)
- Baseline eGFR Category 1 (eGFR<30 mL/min/1.73m2, eGFR ≥30 and <45 mL/min/1.73 m2, eGFR ≥45 mL/min/1.73 m2)
- Baseline eGFR Category 2 (eGFR<15 mL/min/1.73m2, eGFR ≥15 and <30 mL/min/1.73 m2, eGFR ≥30 and <60 mL/min/1.73 m2, eGFR ≥60 and <90 mL/min/1.73 m2, eGFR ≥90 mL/min/1.73 m2)
- Baseline UPCR (g/g) and UACR (g/g)
- Baseline UPCR Category (<1.5 g/g, and ≥ 1.5 g/g)
- Baseline Proteinuria (g/day)
- Baseline Proteinuria Category (< 2 g/day, ≥ 2 and < 3.5 g/day, and ≥ 3.5 g/day)
- Baseline Gd-IgA1 (μg/L), IgA (mg/dL), IgG (mg/dL), IgM (mg/dL), C3 (mg/dL), C4 (mg/dL), APRIL (μg/L), and BAFF (ng/L)
- Baseline systolic blood pressure (mmHg)

Docusign Envelope ID: E39561FE-8723-4250-B198-4822CBF9C70B

- Baseline diastolic blood pressure (mmHg)
- Duration from IgAN diagnosis (years)
- Time Since Archived Biopsy (years)
- Use of SGLT2 inhibitor at Baseline
- Use of RAASi Medication at Baseline by subcategories (No RAASi, ACEi Only, ARB Only, MRA Only, or Combination)
- Number of Anti-hypertension Medication(s) Used at Baseline
- Anti-hypertension Medication Used at Baseline by Class: ACEi, ARB, MRA, Diuretic, Calcium Channel Blocker, Beta Blocker, and Renin Inhibitors

Demographic and baseline characteristics relative to DB baseline and relative to OLE baseline will be summarized separately with descriptive statistics or counts and percentages of subjects as appropriate by treatment and in total using the ITT Population in OLE Week 96 and final analyses.

3.3.5 Medical History

Medical history will be coded to system organ class and preferred term using the Medical Dictionary for Regulatory Activities (MedDRA). Counts and percentages of subjects with medical history by system organ class and preferred term will be summarized by treatment and in total using the ITT Population.

3.3.6 Prior and Concomitant Medications

Concomitant medications will be coded to anatomical therapeutic chemical (ATC) class and preferred term using the World Health Organization (WHO) Drug Dictionary. For summary purposes, medications will be considered prior medications if they stopped prior to the first dose of study drug and concomitant medications if they were taken at any time after the first dose of study drug (i.e., started prior to the first dose of study drug and were ongoing or started after the first dose of study drug).

In the case that a medication has incomplete start or stop dates and it cannot be determined whether the medication should be considered prior or concomitant, dates will be imputed as described in Section 3.1.5.

Counts and percentages of subjects taking prior and concomitant medications by ATC class and preferred term will be summarized by treatment and in total using the Safety Population.

3.3.7 Study Drug Exposure and Compliance

Days of exposure to study drug will be defined for DB period, OLE period, and the entire study as follows:

Days of exposure during the DB period = date of the last dose during the DB period – date of the first dose during the DB period + 7.

Docusign Envelope ID: E39561FE-8723-4250-B198-4822CBF9C70B

Days of exposure during the OLE period = date of the last dose during the OLE period – date of the first dose during the OLE period + 7.

Days of exposure during the entire study = date of the last dose during the DB period or the OLE period (whichever is later)– date of the first dose during the DB or the OLE period (whichever is earlier) + 7.

Note that the exposure calculation is intended to describe the length of time a subject was exposed to study drug and therefore does not take study drug interruptions into account. Days of exposure to study drug will be summarized by treatment using the Safety Population. Descriptive statistics and counts and percentages of subjects with exposure in the following categories will be presented:

- Double-Blind Period
  - ≤12 weeks (≤84 days)
  - >12 - ≤24 weeks (>84 to ≤168 days)
  - >24 - ≤36 weeks (>168 to ≤252 days)
  - >36 weeks (>252 days)
- Open-Label Extension Period
  - ≤12 weeks (≤84 days)
  - >12 - ≤24 weeks (>84 to ≤168 days)
  - >24 - ≤36 weeks (>168 to ≤ 252 days)
  - >36 - ≤48 weeks (>252 to ≤ 336 days)
  - >48 - ≤60 weeks (>336 to ≤420 days)
  - >60 weeks (>420 days)
- Entire Study Period
  - ≤12 weeks (≤84 days)
  - >12 - ≤24 weeks (>84 to ≤168 days)
  - >24 - ≤36 weeks (>168 to ≤252 days)
  - >36 - ≤48 weeks (>252 to ≤336 days)
  - >48 - ≤60 weeks (>336 to ≤420 days)
  - >60 - ≤72 weeks (>420 to ≤504 days)
  - >72 - ≤84 weeks (>504 to ≤588 days)
  - >84 - ≤ 96 weeks (>588 to ≤672 days)
  - > 96 weeks (>672 days)

Percent compliance to study drug regimen will be defined for DB period, OLE period, and the entire study. Percent compliance will be calculated as 100 x number of actual drug syringes injected/number of expected drug syringes injected. The number of actual and expected drug syringes injected will be calculated as follows:

Docusign Envelope ID: E39561FE-8723-4250-B198-4822CBF9C70B

1. For the DB period, the number of actual syringes injected will be the number of records in “Drug Administration” eCRF marked “Yes” for question “was study drug administrated?” and with unique date of injection prior to the first dose of open-label drug. The number of syringes expected will be the ceiling of {(the actual week 36 visit date – the first DB dose date + 1) / 7}.
2. For the OLE period, the number of actual syringes injected will be the number of records in “Drug Administration” eCRF marked “Yes” for question “was study drug administrated?” and with unique date of injection on or after the actual week 36 visit date. The number of syringes expected will be the ceiling of {(the potential last dose date in OLE – the first dose date in OLE + 1) / 7}, where the last dose date in OLE is the last EC record date on or after the actual week 36 visit date.
3. For the entire study, the number of actual syringes injected will be the number of records in “Drug Administration” eCRF marked “Yes” for question “was study drug administrated?” with a unique date of injection. The number of syringes expected will be the ceiling of {(the last dose date – the first dose date + 1) / 7}, where the last dose date is the last EC record date.

Descriptive statistics of percent compliance of DB period, OLE period, and the entire study will be presented. In addition, counts and percentages of subjects with compliance in the following categories <80%, 80% to 100% and >100% will be presented.

Data listings will be generated for: (1) study drug accountability (including the number of syringes dispensed and returned), (2) study drug compliance (including number of actual syringes injected and number of expected syringes injected), and study drug exposure, and (3) study drug administration (including number of injections, date and time of injection, location of injection performed, and any injection site reaction).

3.4 Efficacy Assessments

Efficacy data will be summarized by randomized treatment using the FAS Population. Unless otherwise indicated, the FAS Population is the primary analysis population for efficacy endpoints.

3.4.1 Primary Efficacy Endpoint

The primary efficacy endpoint is the change from baseline at Week 24 in natural log transformed UPCR based on 24-hour urine sample.

The Primary Endpoint Analysis (Week 24 data cut) was conducted when all subjects completed their Week 24 visits or prematurely discontinued the study. In the Phase 2b Version 1 SAP, the primary efficacy endpoint was pre-specified to be analyzed by MMRM model including UPCR data up to week 36 for all subjects at the time of the week 24 data cut (i.e., partial week 36 UPCR data were included in the model). Formal statistical comparisons were performed to compare the percentage change in UPCR between treatments at each visit up to week 36.

Docusign Envelope ID: E39561FE-8723-4250-B198-4822CBF9C70B

In the Phase 2b Version 2 SAP, the MMRM modeling approach of Week 24 UPCR data has been updated to include UPCR data up to Week 24 for the OLE Week 96 and Final Analyses. Including UPCR data up to Week 24, enables estimation of the true treatment effect of atacicept on UPCR up to Week 24. Formal statistical comparisons will be performed for the percentage change in UPCR between treatments at each visit up to Week 24.

The primary analysis of UPCR data will be based on MMRM analysis. UPCR data will be analyzed following natural log transformation. Change from baseline in log transformed UPCR will be the dependent variable. Fixed effects for randomized treatment, natural log transformed baseline value, baseline eGFR category (<45 mL/min/1.73 m2, eGFR ≥45 mL/min/1.73 m2), visit (as a categorical variable), and treatment-by-visit interaction will be included as independent variables, and subject will be included as a random effect. An unstructured covariance matrix will be used to model the within-subject correlation of data. If it fails to converge, other structures will be used (such as Toeplitz, AR(1), or compound symmetric). The Kenward-Roger's degrees-of-freedom adjustment will be used. Restricted maximum likelihood will be used to obtain parameter estimates. Prior to natural log transformation, baseline value for UPCR will be derived as the geometric mean of all measurements prior to the first dose of study drug.

The primary comparison will evaluate the mean difference in change from baseline in natural log transformed UPCR between the combined 75 mg and 150 mg atacicept groups versus the placebo group at Week 24. This statistical comparison will be based on linear contrasts of the Least Squares Means (LS Means) estimated from the MMRM analysis. The geometric least-squares means, standard errors, the 2-tailed 95% confidence intervals (CIs), and the 2-sided p-values at Week 24 for combined atacicept group and placebo group and for treatment comparison will be presented. Geometric least squares mean values will be obtained by exponentiating the LS Means from the model. The percentage reduction from baseline (adjusted for baseline) estimates will be provided as 1 – Geometric LS Mean (%). For percentage reduction versus Placebo, estimates will be provided for 1 – Ratio of Geometric LS Means (%).

The analysis will be done using the FAS Population.

A separate MMRM model will be conducted to compare the treatment effect between each individual dose (150 mg, 75 mg, and 25 mg) versus placebo and between 150 mg versus 75 mg at Week 24.

The sample SAS code for primary efficacy endpoint analysis can be found in Appendix 2 of this SAP.

3.4.2 Secondary Efficacy Endpoint

The secondary efficacy endpoint is the change from baseline at Week 36 in natural log transformed UPCR.

The End of Part A Analysis (Week 36 data cut) was conducted when all subjects completed their Week 36 visits or prematurely discontinued the study. In Phase 2b Version 1 SAP, the

Docusign Envelope ID: E39561FE-8723-4250-B198-4822CBF9C70B

secondary efficacy endpoint was pre-specified to be analyzed by MMRM model including UPCR data up to Week 36 for all subjects at the time of the week 36 data cut (i.e., completed week 36 UPCR data were included in the model). Formal statistical comparisons were performed to compare the percentage change in UPCR between treatments at each visit up to week 36.

At the time of OLE Week 96 and the Final Analyses, the same analysis (i.e., MMRM model including UPCR data up to Week 36 for all subjects) will be repeated.

The geometric least-squares means, standard errors, the 2-tailed 95% CIs, and the 2-sided p-values at Week 36 for the combined 75 mg and 150 mg atacicept group and placebo group and for treatment comparison will be presented based on the same model stated in Section 3.4.1.

A separate MMRM model will be conducted to compare the treatment effect between each individual dose (150 mg, 75 mg, and 25 mg) versus placebo and between 150 mg versus 75 mg at Week 36.

3.4.3 Sensitivity Analysis

To assess the robustness of the primary and secondary endpoint results, sensitivity analyses will be performed.

3.4.3.1 Sensitivity Analysis Using the PP Population

For the primary efficacy endpoint, the MMRM analyses stated in Section 3.4.1 will be repeated using the Week 24 PP Population.

For the secondary efficacy endpoint, the MMRM analyses stated in Section 3.4.2 will be repeated using the Week 36 PP Population.

3.4.3.2 Missing Data Sensitivity Analysis: Tipping Point

Following Version 1 SAP specifications, a two-dimensional tipping point analysis was performed to assess the robustness of the primary endpoint analysis at Week 24 analysis. Because the UPCR data up to Week 24 were quite complete and the tipping point analysis found missing data had little effect on atacicept treatment effect on UPCR results, the tipping point analysis of the primary endpoint analysis will not be repeated in the OLE Week 96 and Final analyses.

3.4.4 Exploratory Endpoints

3.4.4.1 Change from Baseline in Natural Log Transformed UPCR

<u>Analysis for Atacicept Treatment Period:</u>

Docusign Envelope ID: E39561FE-8723-4250-B198-4822CBF9C70B

In the OLE Week 96 analysis and Final analysis, MMRM models (similar to the one described in Section 3.4.1, except not including randomized treatment and treatment-by-visit interaction as fixed effects) will be fit to the change from ATRT baseline in natural log transformed UPCR to estimate the log-transformed change from ATRT baseline in UPCR for the Atacicept Treated population at Weeks 12, 24, 36, 48, 60, 72, 84, and 96. The percentage change from ATRT baseline (derived from geometric least-squares means of log-transformed UPCR change), percentage change associated standard errors, the 2-tailed 95% CIs will be presented by visit. No between-treatment comparison will be performed because all subjects will be grouped into 1 group.

Analysis for Entire Study Period (Up to Week 96):

In the OLE Week 96 analysis and Final analysis, MMRM models (similar to the one described in Section 3.4.1) will be fit to the change from baseline in natural log transformed UPCR to estimate the log-transformed change from DB baseline in UPCR for the all atacicept (i.e., includes pooled subjects originally randomized to any atacicept group in the DB period) and placebo groups at Weeks 12, 24, 36, 48, 60, 72, 84, and 96. The percentage change from DB baseline (derived from geometric least-squares means of log-transformed UPCR change), percentage change associated standard errors, the 2-tailed 95% CIs for the all atacicept and placebo groups will be presented by visit. No between-treatment comparison will be performed because all subjects receive the same treatment (open label atacicept 150 mg SC QW) from Week 36 to Week 96.

3.4.4.2 Change from Baseline in Natural Log Transformed eGFR

Analysis for Double-Blinded Period (Up to Week 36):

Phase 2b Version 1 SAP pre-specified that the change from baseline in natural-log-transformed eGFR be analyzed by MMRM including eGFR data up to Week 36 for all FAS subjects at the time of the Week 24 and Week 36 data cuts. Formal statistical comparisons were performed to compare the percentage change in eGFR between treatments at each visit up to Week 36.

For OLE Week 96 and Final Analyses, the same analysis (i.e., MMRM model including eGFR data up to Week 36 for all subjects) will be repeated.

The MMRM model will be constructed similarly to the MMRM model used to analyze the change from baseline in natural log transformed UPCR (as described in Section 3.4.1), except natural log-transformed baseline eGFR will be used to replace the natural log-transformed baseline UPCR in the model, and baseline eGFR category (<45 mL/min/1.73 m2, eGFR ≥45 mL/min/1.73 m2) will be removed to avoid overfitting the model. The first MMRM model will estimate the treatment effect for combined atacicept 150 mg and 75 mg group vs. placebo at each postbaseline visit up to Week 36. The second MMRM model will estimate the treatment effect for each individual atacicept dose vs. placebo at each postbaseline visit up to Week 36. The geometric least-squares means, standard errors, and the 2-tailed 95% confidence intervals (CIs) for each treatment will be presented at each postbaseline visits up to Week 36, where the geometric least squares mean

Docusign Envelope ID: E39561FE-8723-4250-B198-4822CBF9C70B

values will be obtained by exponentiating the LS Means from the model. The percentage change from baseline estimates will be estimated by (Geometric LS Mean - 1)*100%. The percentage change versus placebo, the corresponding standard error, the 95% CIs, and p-value for the between treatment comparisons will be presented as well. For percentage change versus placebo, estimates will be provided by (Ratio of Geometric LS Means - 1)*100%.

Analysis for Atacicept Treatment Period:

A MMRM model, similar to the MMRM model described above, will be used to estimate the change from ATRT baseline in log-transformed eGFR to all postbaseline visits up to Week 96 for Atacicept Treated population. Since all subjects who received at least 1 dose of either DB or OL atacicept will be pooled into 1 group (i.e., Atacicept Treated group), randomized treatment and treatment-by-visit interaction will be excluded as the fixed effects from the model. All statistics described in the above paragraph will be displayed, except that the p-value for the between-treatment group comparison will not be generated as all subjects will be pooled into 1 group.

Analysis for Entire Study Period (Up to Week 96):

In addition, another MMRM model will be used to estimate the change from DB baseline in log-transformed eGFR to all postbaseline visits up to Week 96. This model will include two treatment groups (all atacicept and placebo). All statistics described in the above paragraph will be displayed, except that the p-value for the between-treatment group comparison will not be generated as all subjects received the same treatment (open label atacicept 150 mg) from Week 36 to Week 96.

The mean absolute change from baseline will be further calculated by baseline geometric mean for all subjects *times the percentage change from baseline in each treatment group.

3.4.4.3 Change from Baseline in eGFR Using the Original Scale

Change from baseline in eGFR using the original scale will be included as an additional exploratory endpoint in the Phase 2b Version 2 and Version 3 SAPs.

The following analysis will be conducted in the OLE Week 96 and the Final Analyses:

Docusign Envelope ID: E39561FE-8723-4250-B198-4822CBF9C70B

Analysis for Atacicept Treatment Period:

A MMRM model will be fit to the change from ATRT baseline in eGFR on original scale including data up to Week 96 (in both the OLE Week 96 and the Final Analyses) for Atacicept Treated population. The MMRM model will be conducted similarly to the MMRM model used to analyze the change from baseline in natural log transformed eGFR, except the change from ATRT baseline on original scale in eGFR will be the dependent variable. The baseline eGFR value on original scale and visit (as a categorical variable) will be included as fixed independent variables, and subject will be included as a random effect. The least-squares means, standard errors, the 2-tailed 95% CIs for each visit will be presented. Since all subjects who received at least 1 dose of either DB or OL atacicept will be pooled into 1 group (i.e., Atacicept Treated group), randomized treatment and treatment-by-visit interaction will be excluded as the fixed effects and no between-treatment comparison will be performed.

Analysis for Entire Study Period:

Another MMRM model will be fit to the change from baseline in eGFR using the original scale including data up to Week 96 (in both the OLE Week 96 and the Final Analyses) and up to the safety follow-up +26 weeks (only in the Final Analysis) for FAS population. The MMRM model will estimate the treatment effect for the all atacicept and placebo groups at each postbaseline visit. The MMRM model will be constructed similarly to the MMRM model used to analyze the change from baseline in natural log transformed eGFR, except the change from baseline using the original scale in eGFR will be the dependent variable. Fixed effects for randomized treatment, baseline eGFR value using the original scale, visit (as a categorical variable), and treatment-by-visit interaction will be included as independent variables, and subject will be included as a random effect. The least-squares means, standard errors, the 2-tailed 95% CIs for each treatment or pooled treatment will be presented by visit. No between-treatment comparison will be performed because all subjects receive the same treatment (open label atacicept 150 mg) from Week 36 to Week 96.

3.4.4.4 Annualized rate of change in eGFR through Week 96

The annualized rate of change in eGFR estimated by mixed-effect-model random coefficients analysis was a pre-specified analysis in protocol Version 4. The Phase 2b Version 1 SAP clarified that the annualized rate of change in eGFR was not estimated in the Primary Endpoint Analysis at Week 24 and the End of Part A Analysis at Week 36, since the follow-up period was too short to have a reliable estimate of eGFR slope. The annualized rate of change in eGFR will be estimated in the OLE Week 96 and the Final Analyses.

Analysis for Atacicept Treatment Period:

To estimate the annualized total slope for eGFR for Atacicept Treated population, a mixed-effects model with random intercept and random slope (MMRIRS) will be constructed to analyze the rate of change in eGFR including (1) all eGFR data up to Week 96 for subjects originally randomized to active atacicept groups and (2) all eGFR data after the first dose of OL atacicept up to Week 96 for subjects originally randomized to placebo. The MMRIRS

Docusign Envelope ID: E39561FE-8723-4250-B198-4822CBF9C70B

model will be used to estimate the annualized total slope for the Atacicept Treated group through Week 96, where it is assumed that the rate of change in eGFR is the same before and after switching to open label atacicept and the same rate of change in eGFR across the different dose groups. In the random coefficient model, eGFR on original scale will be the dependent variable, with random subject effects for intercepts and slopes, and fixed effects for baseline eGFR and time. The actual time measurements, in years after the first injection (i.e., total slope) of either DB or OL atacicept, will be included in the model as a continuous variable. The baseline eGFR value recorded per subject will be assigned a time value of 0. The least-squares means estimate for the coefficient of time will be used to estimate the annualized rate of change in eGFR, its corresponding standard errors, the 2-tailed 95% CIs for the Atacicept Treated group will also be presented.

<u>Analysis for Entire Study Period (up to Week 96):</u>

To estimate the annualized total slope for eGFR for the all atacicept group, a mixed-effects model with random intercept and random slope (MMRIRS) will be constructed to analyze the rate of change in eGFR including all eGFR data for all subjects originally randomized to any atacicept group up to Week 96 (i.e., Excluding eGFR data collected in placebo group or safety follow-up visits). The MMRIRS model will be used to estimate the annualized total slope for the all atacicept group through Week 96, where it is assumed that the rate of change in eGFR is the same before and after switching to open label atacicept and the same rate of change in eGFR across the different dose groups. In the random coefficient model, eGFR will be the dependent variable, with random subject effects for intercepts and slopes, and fixed effects for baseline eGFR and time. The actual time measurements, in years after the first injection (i.e., total slope), will be included in the model as a continuous variable. The baseline eGFR value recorded per subject will be assigned a time value of 0. The least-squares means estimate for the coefficient of time will be used to estimate the annualized rate of change in eGFR, its corresponding standard errors, the 2-tailed 95% CIs for the all atacicept group will also be presented.

3.4.4.5 Hematuria Resolution and Hematuria Improvement

<u>Analysis for Entire Study Period (up to Week 96):</u>

Hematuria resolution and hematuria improvement will be included as exploratory endpoints in Phase 2b Version 2 SAP.

Hematuria categories are defined based on urine dipstick (labelled as “occult blood” in laboratory database) results into 4 categories: Negative/Trace = Negative or 0.03; 1+ = 0.06 or 0.1; 2+ = 0.2 or 0.5; and 3+ = 1.0 or >1.0.

Hematuria resolution is defined as hematuria improvement to Negative/Trace from ≥1+ at baseline. Hematuria improvement is defined as hematuria decreased at least 1 category in those with hematuria 1+ to 3+ at baseline (for example, the hematuria changing from 1+ at baseline to Negative/Trace at Week 12, or hematuria changing from 3+ at baseline to 2+ at Week 36 would be considered improvement).

Docusign Envelope ID: E39561FE-8723-4250-B198-4822CBF9C70B

The following analyses will be conducted in the OLE Week 96 Analysis and the Final Analysis:

- The number and percentage of subjects with and without hematuria resolution, respectively, by visit and treatment group for subjects with 1+ to 3+ hematuria at baseline.
- The number and percentage of subjects with and without hematuria improvement, respectively, by visit and treatment group for subjects with 1+ to 3+ hematuria at baseline.

For both analyses, only subjects with 1+ to 3+ hematuria at baseline will be included in this analysis. For each postbaseline visit, the number of subjects with non-missing hematuria results will be included and used as the denominator for percentage calculation. The corresponding 95% CI will be estimated from the exact (Clopper-Pearson) CI for the binomial proportion. The p-value for comparing the proportion of subjects with hematuria resolution/improvement in individual atacicept groups and all atacicept group versus placebo will be estimated from the Fisher exact method for visits occurring in the DB period only.

In addition, the number and percentage of subjects in each hematuria category will be summarized by visit and treatment group for the FAS population. The change from baseline in percentage of subjects with hematuria 1+ to 3+ among those with baseline hematuria 1+ to 3+ will also be summarized by visit and treatment group.

<u>Analysis for Atacicept Treatment Period:</u>

Similar analyses will be repeated by visit (relative to ATRT baseline) for Atacicept Treated population after they received the first dose of either DB or OL atacicept.

3.4.4.6 Change from Baseline in Natural Log Transformed UACR

<u>Analysis for Double-Blinded Period:</u>

The Phase 2b Version 1 SAP pre-specified the change from baseline in natural log-transformed UACR be analyzed by MMRM including UACR data up to Week 36 for all subjects at the time of the Week 24 and Week 36 data cuts. Formal statistical comparisons were performed to compare the percentage change in UACR between treatments at each visit up to Week 36.

At the time of the OLE Week 96 and the Final Analyses, the same analysis (i.e., MMRM model including UACR data up to Week 36 for all subjects) will be repeated.

Two MMRM models will be used to analyze the mean change from baseline over time for UACR using natural log-transformations based on the same model stated in Section 3.4.1, except natural log-transformed baseline UACR will be used to replace the natural log-transformed baseline UPCR in the model. The first MMRM model will estimate the treatment effect and treatment comparison for combined atacicept 150 mg and 75 mg group vs. placebo at each postbaseline visit up to Week 36. The second MMRM model will

Docusign Envelope ID: E39561FE-8723-4250-B198-4822CBF9C70B

estimate the treatment effect and treatment comparison for each individual atacicept group vs. placebo at each postbaseline visit up to Week 36.

In addition, the following analyses will be conducted in the OLE Week 96 analysis and the Final analysis.

<u>Analysis for Atacicept Treatment Period:</u>

A MMRM model (similar to the one described in Section 3.4.4.1, except not including randomized treatment and treatment-by-interaction term as the fixed effects) will be fit to the change from ATRT baseline in natural log-transferred biomarker including data up to Week 96 for Atacicept Treated population. The MMRM model will be used to estimate the treatment effect for Atacicept Treated subjects at each postbaseline visit. The geometric least-squares means, standard errors, the 2-tailed 95% CIs will be presented by visit. No between-treatment comparison will be performed because all subjects will be pooed into 1 group (i.e., Atacicept Treated group).

<u>Analysis For Entire Study Period (up to Week 96):</u>

A MMRM model (similarly as described in Section 3.4.4.1) will be fit to the change from baseline in natural log-transferred UACR at all postbaseline visits up to Week 96. The MMRM model will estimate the treatment effect for the all atacicept group and placebo at each postbaseline visit up to Week 96. The geometric least-squares means, standard errors, the 2-tailed 95% CIs for the all atacicept group and placebo will be presented by visit. No between-treatment comparison will be performed because all subjects receive the same treatment (open label atacicept 150 mg) from Week 36 to Week 96.

3.4.4.7 Change from Baseline in Biomarkers: Gd-IgA1, IgA, IgG, and IgM

<u>Analysis for Double-Blinded Period:</u>

The Phase 2b Version 1 SAP pre-specified the change from baseline in natural log-transformed biomarker vbe analyzed by MMRM including biomarker data up to Week 36 for all subjects at the time of the Week 24 and Week 36 data cuts. Formal statistical comparisons were performed to compare the percentage change in biomarker between treatments at each visit up to Week 36.

At the time of the OLE Week 96 and Final Analyses, the same analysis (i.e., MMRM model including biomarker data up to Week 36 for all subjects) will be repeated.

For each biomarker endpoint, the MMRM model will be used to analyze the mean percentage change from baseline in biomarker over time based on the same model stated in Section 3.4.1, except natural log-transformed baseline in the biomarker under evaluation will be used to replace the natural log-transformed baseline UPCR in the model. The MMRM model will estimate the treatment effect and treatment comparison for each individual atacicept group vs. placebo at each postbaseline visit up to Week 36.

Docusign Envelope ID: E39561FE-8723-4250-B198-4822CBF9C70B

In addition, the following analyses will be conducted in the OLE Week 96 Analysis and the Final Analysis for each biomarker separately.

Analysis for Atacicept Treatment Period:

A MMRM model (similar to the one described in Section 3.4.4.1, except not including randomized treatment and treatment-by-interaction term as fixed effects) will be fit to the change from ATRT baseline in natural log-transferred biomarker including data up to Week 96 for Atacicept Treated population. The MMRM model will be used to estimate the treatment effect for atacicept treated subjects at each postbaseline visit. The geometric least-squares means, standard errors, the 2-tailed 95% CIs will be presented by visit. No between-treatment comparison will be performed because all subjects will be pooed into 1 group (i.e., Atacicept Treated group).

Analysis for Entire Study Period:

A MMRM model (similar to the one described in Section 3.4.4.1) will be fit to the change from baseline in natural log-transferred biomarker including data up to Week 96 (in both the OLE Week 96 and the Final Analyses) and up to safety follow-up +26 weeks (only in the Final Analysis). The MMRM model will be used to estimate the treatment effect for the all atacicept group and placebo at each postbaseline visit. The geometric least-squares means, standard errors, the 2-tailed 95% CIs for the all atacicept group and placebo will be presented by visit. No between-treatment comparison will be performed because all subjects receive the same treatment (open label atacicept 150 mg) from Week 36 to Week 96.

3.4.4.8 Absolute Change from Baseline and Percentage Change from Baseline for UPCR, eGFR, Gd-IgA1, IgA, IgG, IgM, C3 and C4 level

Absolute, change from DB baseline, and percentage change from DB baseline at all post-baseline visits for UPCR, eGFR, Gd-IgA1, IgA, IgG, IgM, C3 and C4 levels will be summarized by treatment group with descriptive statistics using the FAS population.

For the OLE Week 96 and the Final Analyses, ATRT baseline, change from ATRT baseline, and percentage change from ATRT baseline by visits up to Week 96 for selected efficacy endpoint will be summarized with descriptive statistics using the Atacicept Treated population.

For the OLE Week 96 and the Final Analyses, OLE baseline, change from OLE baseline, and percentage change from OLE baseline at all OLE and safety follow-up visits for each efficacy endpoint will be summarized by treatment group with descriptive statistics using the FAS population.

3.4.4.9 Serum Concentration of Atacicept

Per protocol, on Day 1, following monitoring of any ISR, a blood sample was to be collected between 2-4 hours after the first dose. After Day 1, at PK timepoints in the Schedule of Assessments, a blood sample was to be collected prior to administration of study drug. Blood

Docusign Envelope ID: E39561FE-8723-4250-B198-4822CBF9C70B

samples for PK analysis to assess trough levels of serum atacicept was to be collected for all subjects.

The concentrations of atacicept will be listed for each subject in the Final Analysis.

3.4.4.10 Atacicept Anti-Drug Antibodies

OLE Week 96A positive ADA sample is a sample with ADA assay screening results as “putative positive” and ADA assay confirmatory result as “positive”. The number and percentage of subjects who develop detectable positive ADAs will be summarized by treatment groups as follows:

- Subjects with pre-existing positive ADA at Baseline and with a least one valid and positive ADA sample after atacicept initiation labelled as “Pre-Existing ADA positive”
- Subjects without pre-existing positive ADA at Baseline and with at least one valid and positive ADA sample after atacicept initiation labelled as “Treatment-emergent ADA positive”

ADA titers will be summarized descriptively for ADA positive samples. If an ADA assay confirmatory result is positive, the confirmed positive samples may be assayed in the future for neutralizing antibody activity.

An ADA summary will be included in the Final Analysis.

3.5 Safety Assessments

Safety data will be summarized by study drug administered using the Safety Population.

3.5.1 Adverse Events (AEs)

AEs will be captured from the date of informed consent through study completion. All AEs will be coded to system organ class and preferred term using the MedDRA dictionary. The version of MedDRA dictionary used will be included in the footnote for relevant summary tables.

Treatment emergent adverse events (TEAEs) during the study are defined as TEAEs that start on or after the first dose of study drug and will be assigned to the most recently received treatment at the start of the TEAE.

TEAEs during the DB period will include AEs started on or after the first dose of DB study drug through the completion of the DB period or prior to the first dose of study drug in the OLE period.

TEAEs during the OLE period will include AEs started on or after the first dose of OLE study drug through the end of the study.

TEAEs during the entire study period will include AEs started on or after the first dose of DB study drug through the end of the study.

TEAEs during the atacicept treatment period will include AEs start on or after the first dose of atacicept (in, the first dose of DB atacicept for subjects originally randomized to atacicept groups or the first dose of open label atacicept 150 mg for subjects originally randomized to placebo group) through the last dose of atacicept plus 30 days.

TEAEs will be summarized separately during the DB period, the OLE period, during the entire study period, and during the atacicept treatment period, as appropriate.

An overview of TEAEs will be provided including counts and percentages of subjects with the following:

- Any TEAEs (overall and by maximum severity)
- Any study drug related TEAEs (overall and by maximum severity)
- Any serious AEs (SAEs)
- Any treatment-emergent serious AEs (TESAEs)
- Any TEAEs of special interest defined by relevant SMQ narrow scope search (overall and by maximum severity)
- Any injection site reaction (ISR) TEAEs defined by injection site reaction PT or by combined PTs (overall and by maximum severity)
- Any protocol defined special situation TEAEs
- Any TEAEs leading to premature discontinuation of study drug
- Any TEAEs leading to premature discontinuation of study
- Any AEs with an outcome of death

Frequency counts and percentages of subjects with TEAEs will be presented by system organ class and preferred term for TEAEs, study drug related TEAEs, serious TEAEs, and TEAEs of special interest. A subject who experienced multiple TEAEs within a system organ class (SOC) or preferred term (PT) will be counted only once for the SOC or PT. For tabulations that include classification by relationship to study treatment, TEAEs with missing relationship will be considered related to study drug.

Subjects with any TEAEs, study drug-related TEAEs, and TEAEs of special interest will be tabulated by SOC, PT, and maximum severity. TEAEs with missing severity will be included in a missing category and will be considered as the least severe category.

Adverse events of special interest (AESIs) include hypersensitivity reactions, opportunistic infections, cardiac disease/disorders (specifically cardiac failure, cardiac arrhythmia, and ischemic heart disease), and demyelination disorders. AESIs will be identified by two approaches. For analysis purposes, a list of preferred terms from relevant Standardized MedDRA Query (SMQ) narrow scope will be used to flag AESIs in a more systematic and objective way. AESIs identified by SMQ search will be included in a tabulation summary.

Docusign Envelope ID: E39561FE-8723-4250-B198-4822CBF9C70B

Site investigators are asked to identify any AEs meeting the AESI criteria and report them. AESI identified by both approaches will be identified in listings.

Listings will be presented for SAEs, AESIs, TEAEs leading to premature discontinuation of study drug, TEAEs leading to premature discontinuation of study and SAEs with an outcome of death.

3.5.2 Clinical Laboratory Tests

Blood and urine samples will be collected for clinical laboratory tests at the timepoints listed in Table 1. Summary statistics will be provided for safety laboratory tests at DB baseline and all scheduled post-baseline visits for chemistry, hematology, urinalysis, and other tests by treatment group. Values and changes from DB baseline will be presented at each scheduled visit for chemistry, hematology, urinalysis, and other laboratory tests by treatment group using the FAS population. The incidence of abnormalities (as defined by normal ranges) prior to the first dose of study drug and after the first dose of study drug will be summarized with counts and percentages of subjects.

In addition, for the OLE Week 96 and the Final Analyses, OLE baseline, absolute values, and changes from OLE baseline at all OLE and safety follow-up visits for each numerical laboratory test will be summarized by treatment group with descriptive statistics using the FAS population.

3.5.3 Vital Signs

Vital sign measurements (systolic and diastolic blood pressure, pulse rate, respiratory rate, temperature), weight and height (at screening only) will be measured prior to any other study-related procedures, at the scheduled visits. Vital sign DB baseline, absolute values, and change from DB baseline will be summarized by timepoint and treatment group with descriptive statistics using the FAS population.

In addition, for the OLE Week 96 and the Final Analyses, OLE baseline, values, and changes from OLE baseline for each vital sign will be summarized by timepoint and treatment group with descriptive statistics using the FAS population.

3.5.4 Electrocardiograms (ECG)

Digital ECGs will be recorded by study sites using an appropriate ECG machine. A standard, single, digital 12-lead ECG will be obtained as outlined in Table 1. Counts and percentages of ECG overall interpretation results with normal, abnormal (further categorized as clinically significant abnormal vs. not clinically significant abnormal), indeterminate, not evaluable, and unknown categories will be summarized.

3.5.5 Physical Examinations

A complete physical examination will include, at a minimum, assessment of the General Appearance, Skin, Lymph Nodes, Head, Ears, Eyes, Nose, Throat, Neck, Thorax/Lungs, Cardiovascular, Abdomen, Musculoskeletal, and Neurological systems. Complete physical examination will be performed at screening, week 24, and week 96, and ET visits. On other visits, a symptom-driven physical examination will be performed, and will include assessment of any new subject complaints or changes from baseline. Whether there is any clinically significant finding (Yes or No) during the physical examination will be evaluated as outlined in Table 1. As physical examination findings are qualitative and there is significant inter-observer variation, no analyses will be performed on the physical examination data. All physical examination findings will be presented in a listing.

3.5.6 EuroQol (EQ-5D-5L) Study Questionnaire

The EQ-5D-5L consists of the EQ-5D descriptive system and the EQ visual analog scale (EQ VAS).

- The EQ-5D questionnaire comprises 5 questions (items) relating to current problems in the dimensions: mobility, self-care, usual activities, pain/discomfort, and anxiety/depression. Each dimension has 5 levels: no problems, slight problems, moderate problems, severe problems, and extreme problems.
- The EQ VAS records the subject's self-rated health on a vertical visual analog scale, where the endpoints are labeled 'The best health you can imagine' and 'The worst health you can imagine'. The VAS can be used as a quantitative measure of health outcome that reflects the subject's own judgment.

EQ VAS DB baseline values, each post-baseline value, and change from DB baseline at each post-baseline visit will be summarized by treatment group with descriptive statistics.

All EQ-5D-5L assessments will be presented in a data listing.

4 INDEPENDENT DATA MONITORING COMMITTEE

An independent data monitoring committee (iDMC) will monitor the safety and welfare of the study subjects. The iDMC will meet approximately every 6 months to review accumulated safety data and may make recommendations regarding the conduct of the study based on the accumulated data. Vera retains final decision-making authority on all aspects. Details regarding the role, responsibilities, and data review requirements of the iDMC will be outlined in a separate iDMC charter.

Docusign Envelope ID: E39561FE-8723-4250-B198-4822CBF9C70B

5 MID-STUDY ANALYSIS AND FINAL ANALYSIS TIMING

5.1 Week 24 Primary Endpoint Analysis

The primary UPCR endpoint analysis was performed after all subjects completed the Week 24 visit or prematurely discontinued from the study. This analysis was performed using all data when all subjects completed the Week 24 visit or prematurely discontinued from the study, including data beyond Week 24 for subjects that had later timepoint data collected at the time of the analysis. The database was cleaned for all subjects. The primary endpoint analysis at Week 24 was performed and reviewed by an independent CRO's and Sponsor's unblinded statistical team, who was unblinded at the time of analysis, so that the study management team remained blinded to individual subject data. Selected sponsor personnel were unblinded to review the aggregated summary results and they were removed from the study management team. Personnel who actively participated in the study management team remained blinded until study unblind at Week 36. For the primary endpoint analysis at week 24, the data cutoff date was 06 December 2022. All derived analysis datasets included data up to this cutoff date, while raw data may have included data beyond this date until the time of database lock of the primary endpoint analysis data. The data cut-off date was documented in the analysis data programming specifications.

5.2 End of Part A Analysis at Week 36 Data Cut

An end of Part A analysis was performed when all subjects completed the DB portion of the study (Week 36) or prematurely discontinued from the study. After the last subject completed the Week 36 Visit, the database was cleaned, locked, and the study was unblinded for analysis.

5.3 OLE Week 96 Analysis at Week 96 Data Cut

OLE Week 96 analysis will be performed when all subjects complete the OLE week 96 visit or prematurely discontinue from the study. After the last subject completes the OLE Week 96 Visit, the database will be cleaned, locked, and analyzed.

After the End of Part A analysis, the sponsor decided to conduct an OLE Week 96 analysis to evaluate the long-term eGFR effect to inform the sample size re-check for the Phase 3 part of the study.

5.4 Final Analysis

The Final analysis for this study will be performed when all subjects complete the study or prematurely discontinue from the study. The Final analysis will include all data from DB period, OLE period, and the safety follow-up period.

Docusign Envelope ID: E39561FE-8723-4250-B198-4822CBF9C70B

6 CHANGES FROM PROTOCOL-SPECIFIED STATISTICAL ANALYSES

6.1 Changes from Protocol-specific Statistical Analyses in Version 1 SAP

The protocol version 4.0 does not define urine albumin/creatinine ratio (UACR) as one of the endpoints. UACR was added as an exploratory endpoint in Phase 2b Version 1 SAP. Analysis of UACR at Weeks 12, 24, and Week 36 was conducted in a similar fashion to those for UPCR.

The protocol version 4.0 specifies the FAS as consisting of all randomized subjects who have received at least 1 dose of treatment and have at least 1 post-baseline measurement of UPCR. The Full Analysis Set definition was updated as "FAS population consists of all randomized subjects who have received at least 1 dose of treatment" based on comments received by the US Food and Drug Administration.

The protocol specifies that MMRIRS will be used to analyze the rate of change in eGFR. eGFR will be the dependent variable, with random subject effects for intercepts and slopes, and fixed effects for randomized treatment, baseline eGFR, time, and the treatment-by-time interaction. The analysis tests differences between the treatment groups constructed using linear contrasts of the appropriate LS Means from the mixed effects model. However, given the double-blinded nature of the study and the randomized phase lasting for only 36 weeks, after which all subjects switched to open-label atacicept 150 mg, the duration may not have been ideal to provide a reliable estimate for the rate of change in eGFR. As a result, a mixed-model repeated measures analysis (as described for the primary endpoint) was used to analyze the mean change from baseline over time for eGFR using natural log-transformations as detailed in Section 3.4.4.2.

The protocol version 4.0 does not specify baseline eGFR category (<45 mL/min/1.73 m2, eGFR ≥45 mL/min/1.73 m2) as an independent variable for the primary efficacy analysis of UPCR data. The Phase 2b Version 1 SAP specified that baseline eGFR category (<45 mL/min/1.73 m2, eGFR ≥45 mL/min/1.73 m2) would be included as an independent variable in the mixed-model repeated measures analysis for all primary, secondary, and exploratory efficacy endpoints except for eGFR. The mixed-model repeated measures analysis for eGFR excluded baseline eGFR category as an independent variable from the model in order to avoid over fitting the model because the model already included the log-transformed baseline eGFR value as one of the independent variables for eGFR analysis.

6.2 Changes from Version 1 SAP

Version 1 SAP specified two interim analyses (i.e., the Primary Endpoint analysis and the End of Part A analysis) and the Final analysis. After the End of Part A analysis, the Sponsor decided to conduct an OLE Week 96 analysis to evaluate the long-term eGFR effect to inform the sample size re-check for the Phase 3 part of the study.

Text in Section 3.4 (Efficacy Assessment) was revised to clarify how each efficacy endpoint was analyzed in the Primary Endpoint Analysis (using Week 24 data cut) and the End of Part A Analysis (using Week 36 data cut). A new text was added to clarify how each efficacy endpoint will be analyzed in OLE Week 96 Analysis and the Final Analysis.

Docusign Envelope ID: E39561FE-8723-4250-B198-4822CBF9C70B

The exploratory endpoints of "change from baseline by visit in natural log transformed C3 and C4 levels" were removed, since C3 and C4 are not closely tied to Atacicept's Mechanism of Action (MOA) in IgAN.

The protocol does not define hematuria as one of the endpoints. Recent research suggests hematuria could be a meaningful clinical endpoint in trials for IgA nephropathy. As a result, hematuria resolution/improvement was added as new exploratory endpoints (see details in Section 3.4.4.4).

Two new models were added to analyze the long-term eGFR data. The first model is an MMRM model fit to the change from baseline in eGFR using the original scale based on comments from regulatory agencies. This MMRM model will be constructed similarly to the MMRM model used to analyze the change from baseline in natural log transformed eGFR, except the change from baseline using the original scale in eGFR will be the dependent variable. Fixed effects for randomized treatment, baseline eGFR value using the original scale, study visit (as a categorical variable), and treatment-by-visit interaction will be included as independent variables, and subject will be included as a random effect (see details in Section 3.4.4.3). The second model is an MMRIRS model to estimate the annualized total slope for eGFR for all subjects originally randomized to any atacicept group up to Week 96. This MMRIRS model will include eGFR as the dependent variable, random subject effects for intercepts and slopes, and fixed effects for baseline eGFR and time (see details in Section 3.4.4.3).

The plots of mean (SD) absolute changes from baseline and percentage changes from baseline in UPCR, eGFR, Gd-IgA1, IgA, IgG, IgM, C3 and C4 levels based on descriptive statistics will not be produced in the OLE Week 96 and the Final Analyses due to the skew of these parameters.

Serum concentrations of atacicept will not be summarized by timepoint and treatment group with descriptive statistics in the OLE Week 96 and Final analyses. Instead, the concentrations of atacicept will be included as the source data to support population PK modeling and summarized in population PK report.

6.3 Changes from Version 2 SAP

Text in Section 3.4 (Efficacy Assessment) was revised to add new efficacy analyses for "atacicept treatment period". For subjects originally randomized to active atacicept groups and received at least 1 dose of atacicept, all data will be included for this analysis. For subjects originally randomized to placebo group and received at least 1 dose of OLE atacicept, data collected on or after the first dose of OLE atacicept will be included and will be remapped relative to the first dose of OLE atacicept (such as OLE Week 48 will be remapped as Week 12). As a result, subjects originally randomized to active atacicept groups will have a treatment period receiving up to 96 weeks of atacicept, while subjects originally randomized to placebo groups will have a treatment period receiving up to 60 weeks of atacicept. All subjects will then be pooled as a single group for the new efficacy analysis for "atacicept treatment period".

Docusign Envelope ID: E39561FE-8723-4250-B198-4822CBF9C70B

7 PROGRAMMING SPECIFICATIONS

Analyses will be performed using SAS® version 9.3 or higher. All available data will be presented in subject data listings which will be sorted by subject and visit date as applicable. Detailed Programming Specifications will be provided in a separate document.

Docusign Envelope ID: E39561FE-8723-4250-B198-4822CBF9C70B

APPENDICES

Appendix 1: Laboratory Tests

**Laboratory Assessments**	**Parameters**		
Hematology	Hematocrit<br>Hemoglobin<br>Mean corpuscular hemoglobin<br>Mean corpuscular hemoglobin concentration<br>Mean corpuscular volume<br>Platelet count	RBC Indices:<br>MCV<br>MCH<br>%Reticulocytes	WBC Count with Differential:<br>Neutrophils<br>Lymphocytes<br>Monocytes<br>Eosinophils<br>Basophils
Clinical Chemistry	Albumin<br>Alkaline phosphatase<br>Alanine aminotransferase<br>Aspartate aminotransferase<br>Total bilirubin<br>Bilirubin-direct<br>(only if total bilirubin is outside the normal range)	Calcium<br>Serum creatinine<br>(eGFR will be calculated from serum creatinine using the CKD-EPI equation [Levey 2011])	Glucose<br>Potassium<br>Sodium<br>Total protein<br>Uric acid
Urine Sediment Analysis	Urine red blood cells and white blood cells/high powered field<br>Casts<br>Organisms<br>Crystals		
Routine Urinalysis	Specific gravity<br>pH, glucose, protein, blood, ketones, bilirubin, urobilinogen, nitrite, leukocyte esterase by dipstick<br>Microscopic examination (if blood or protein is abnormal).		
Quantitative Analysis (24 -hour Urine Collection)	Total volume<br>Total protein<br>Total albumin<br>Total creatinine	UPCR<br>UACR	
Other Tests	Serum IgG, IgA, IgM<br>Gd-IgA1<br>Complement (C3, C4)<br>Serum BLyS and APRIL level (if assay is available)<br>HBsAg, anti-HBc [total and IgM] (hepatitis B)<br>Anti-atacicept antibody titers (binding and neutralizing)<br>anti-HCV (if anti-HCV positive with reflex testing for HCV RNA by PCR), anti-HIV 1 and 2<br>HbA1c<br>TSH<br>FSH (for women only)<br>Serum pregnancy test (β-hCG) required only for WOCBP at Screening. Local urine testing will be standard at all other visits unless serum testing is required by local regulation or IRB/IEC. Positive urine pregnancy test results will be confirmed by serum pregnancy testing.<br>Quantiferon test for TB<br>Urine drug screen for amphetamines, cocaine, methadone, and opiates		

β-hCG= beta human chorionic gonadotropin; antiHBc=anti-hepatitis B core; anti-HCV = anti-hepatitis C virus; CKD-EPI=Chronic Kidney Disease Epidemiology Collaboration; eGFR=estimated glomerular filtration rate; HbA1c=hemoglobin-A1c; HBsAg=hepatitis B surface antigen; HCV=hepatitis C virus; HIV=human immunodeficiency virus; IEC=Independent Ethics Committee; Ig=immunoglobulin; IRB=Institutional Review Board; MCH=mean corpuscular hemoglobin; MCV=mean corpuscular volume; PCR=polymerase chain reaction; RBC=red blood cell; RNA=ribonucleic acid; TB=tuberculosis; TSH=thyroid stimulating hormone; WBC=white blood cell; WOCBP=woman of childbearing potential.

Docusign Envelope ID: E39561FE-8723-4250-B198-4822CBF9C70B

Appendix 2: SAS Code

The sample SAS code for the primary comparison of UPCR for combined 75 mg and 150 mg atacicept groups vs. placebo at Week 12, Week 24, and Week 36 is provided below:

```
**************************************************************************
* TRTN = Treatment group [1=Placebo, 2=Combined Atacicept]
* USUBJID = Subject ID
* VISIT = Post-baseline study visits
* GFR = Baseline eGFR category [<45 vs. >=45 mL/min/1.73m2]
* UPCR_B = log (baseline UPCR)
* LOG_CHG = Change of logarithmic UPCR at each post-baseline visit
calculated
            as log (AVAL)-UPCR_B where AVAL=UPCR value at each visit
*************************************************************************;
proc mixed data=efficacy (where= (LOG_CHG ne . and
           VISIT in ('Week 12', 'Week 24', ‘Week 36’))) method=reml;
  class TRTN USUBJID VISIT GFR;
  model LOG_CHG = TRTN VISIT GFR UPCR_B TRTN*VISIT /ddfm=kr;
  repeated VISIT/ sub = USUBJID type = un;
  contrast 'Week 12 Effect' TRTN -1 1 TRTN*VISIT -1 0 0 1 0 0;
  estimate 'Week 12 Effect' TRTN -1 1 TRTN*VISIT -1 0 0 1 0 0/cl
alpha=0.05;
  contrast 'Week 24 Effect' TRTN -1 1 TRTN*VISIT  0 -1 0 0 1 0;
  estimate 'Week 24 Effect' TRTN -1 1 TRTN*VISIT  0 -1 0 0 1 0/cl
alpha=0.05;
  contrast 'Week 36 Effect' TRTN -1 1 TRTN*VISIT  0 0 -1 0 0 1;
  estimate 'Week 36 Effect' TRTN -1 1 TRTN*VISIT  0 0 -1 0 0 1/cl
alpha=0.05;
  lsmeans TRTN*VISIT / pdiff cl;
  ods output mixed.LSMeans=LSMeans;
  ods output mixed.Contrasts=Contrasts;
  ods output mixed.Estimates=Estimates;
run;
```

The sample SAS code for the comparison of UPCR between each individual dose versus placebo and between 150 mg versus 75 mg at Weeks 12, 24, and 36 is provided below:

```
**************************************************************************
* TRTN = Treatment group [1=Atacicept 150 mg, 2 =Atacicept 75 mg,
                          3 = Atacicept 25 mg, 4=Placebo]
* USUBJID = Subject ID
* VISIT = Post-baseline study visits
* GFR = Baseline eGFR category [<45 vs. >=45 mL/min/1.73m2]
* UPCR_B = log (baseline UPCR)
* LOG_CHG = Change of logarithmic UPCR at each post-baseline visit
calculated
            as log(AVAL)-UPCR_B where AVAL=UPCR value at each visit
*************************************************************************;
proc mixed data=efficacy (where=(LOG_CHG ne . and
                          VISIT in ('Week 12', 'Week 24', 'Week 36')))
method=reml;
     class TRTN USUBJID VISIT GFR;
```

Docusign Envelope ID: E39561FE-8723-4250-B198-4822CBF9C70B


```
     model LOG_CHG = TRTN VISIT GFR UPCR_B TRTN*VISIT /ddfm=kr;
     repeated VISIT/ sub = USUBJID type = un;
     contrast 'Week 12 Effect 150 mg vs. Placebo'
               TRTN 1 0 0 -1 TRTN*VISIT 1 0 0 0 0 0 0 0 0 -1 0 0;
     estimate 'Week 12 Effect 150 mg vs. Placebo'
           TRTN 1 0 0 -1 TRTN*VISIT 1 0 0 0 0 0 0 0 0 -1 0 0/cl
alpha=0.05;
     contrast 'Week 12 Effect 75 mg vs. Placebo'
               TRTN 0 1 0 -1 TRTN*VISIT 0 0 0 1 0 0 0 0 0 -1 0 0;
     estimate 'Week 12 Effect 75 mg vs. Placebo'
           TRTN 0 1 0 -1 TRTN*VISIT 0 0 0 1 0 0 0 0 0 -1 0 0/cl
alpha=0.05;
     contrast 'Week 12 Effect 25 mg vs. Placebo'
               TRTN 0 0 1 -1 TRTN*VISIT 0 0 0 0 0 0 1 0 0 -1 0 0;
     estimate 'Week 12 Effect 25 mg vs. Placebo'
           TRTN 0 0 1 -1 TRTN*VISIT 0 0 0 0 0 0 1 0 0 -1 0 0/cl
alpha=0.05;
     contrast 'Week 12 Effect 150 mg vs. 75mg'
               TRTN 1 -1 0 0 TRTN*VISIT 1 0 0 -1 0 0 0 0 0 0 0 0;
     estimate 'Week 12 Effect 150 mg vs. 75mg'
           TRTN 1 -1 0 0 TRTN*VISIT 1 0 0 -1 0 0 0 0 0 0 0 0/cl
alpha=0.05;
     contrast 'Week 24 Effect 150 mg vs. Placebo'
               TRTN 1 0 0 -1 TRTN*VISIT 0 1 0 0 0 0 0 0 0 0 -1 0;
     estimate 'Week 24 Effect 150 mg vs. Placebo'
           TRTN 1 0 0 -1 TRTN*VISIT 0 1 0 0 0 0 0 0 0 0 -1 0/cl
alpha=0.05;
     contrast 'Week 24 Effect 75 mg vs. Placebo'
               TRTN 0 1 0 -1 TRTN*VISIT 0 0 0 0 1 0 0 0 0 0 -1 0;
     estimate 'Week 24 Effect 75 mg vs. Placebo'
           TRTN 0 1 0 -1 TRTN*VISIT 0 0 0 0 1 0 0 0 0 0 -1 0/cl
alpha=0.05;
     contrast 'Week 24 Effect 25 mg vs. Placebo'
               TRTN 0 0 1 -1 TRTN*VISIT 0 0 0 0 0 0 0 1 0 0 -1 0;
     estimate 'Week 24 Effect 25 mg vs. Placebo'
           TRTN 0 0 1 -1 TRTN*VISIT 0 0 0 0 0 0 0 1 0 0 -1 0/cl
alpha=0.05;
     contrast 'Week 24 Effect 150 mg vs. 75mg'
               TRTN 1 -1 0 0 TRTN*VISIT 0 1 0 0 -1 0 0 0 0 0 0 0;
     estimate 'Week 24 Effect 150 mg vs. 75mg'
           TRTN 1 -1 0 0 TRTN*VISIT 0 1 0 0 -1 0 0 0 0 0 0 0/cl
alpha=0.05;
     contrast 'Week 36 Effect 150 mg vs. Placebo'
               TRTN 1 0 0 -1 TRTN*VISIT 0 0 1 0 0 0 0 0 0 0 0 -1;
     estimate 'Week 36 Effect 150 mg vs. Placebo'
           TRTN 1 0 0 -1 TRTN*VISIT 0 0 1 0 0 0 0 0 0 0 0 -1/cl
alpha=0.05;
     contrast 'Week 36 Effect 75 mg vs. Placebo'
               TRTN 0 1 0 -1 TRTN*VISIT 0 0 0 0 0 1 0 0 0 0 0 -1;
     estimate 'Week 36 Effect 75 mg vs. Placebo'
           TRTN 0 1 0 -1 TRTN*VISIT 0 0 0 0 0 1 0 0 0 0 0 -1;
     contrast 'Week 36 Effect 25 mg vs. Placebo'
               TRTN 0 0 1 -1 TRTN*VISIT 0 0 0 0 0 0 0 0 1 0 0 -1;
     estimate 'Week 36 Effect 25 mg vs. Placebo'
           TRTN 0 0 1 -1 TRTN*VISIT 0 0 0 0 0 0 0 0 1 0 0 -1/cl
alpha=0.05;
     contrast 'Week 36 Effect 150 mg vs. 75mg'
```

Docusign Envelope ID: E39561FE-8723-4250-B198-4822CBF9C70B


```
               TRTN 1 -1 0 0 TRTN*VISIT 0 0 1 0 0 -1 0 0 0 0 0 0;
     estimate 'Week 36 Effect 150 mg vs. 75mg'
           TRTN 1 -1 0 0 TRTN*VISIT 0 0 1 0 0 -1 0 0 0 0 0 0/cl
alpha=0.05;
     lsmeans TRTN*VISIT / pdiff cl;
     ods output mixed.LSMeans=LSMeans;
     ods output mixed.Contrasts=Contrasts;
     ods output mixed.Estimates=Estimates;
run;
```

The sample SAS code for the comparison of GFR for combined 75 mg and 150 mg atacicept groups vs. placebo at Weeks 2, 4, 12, 24, and 36 is provided below:

```
**************************************************************************
* TRTN = Treatment group [1=Placebo, 2=Combined Atacicept]
* USUBJID = Subject ID
* VISIT = Post-baseline study visits
* LOGBASE = log (baseline GFR)
* LOG_CHG = Change of logarithmic GFR at each post-baseline visit
calculated
            as log(AVAL)-LOGBASE where AVAL=GFR value at each visit
*************************************************************************;
proc mixed data=efficacy (where=(LOG_CHG ne . and
     VISIT in ('Week 2', 'Week 4','Week 12', 'Week 24', ‘Week 36’)))
                method=reml;
     class TRTN USUBJID VISIT;
     model LOG_CHG = TRTN VISIT LOGBASE TRTN*VISIT /ddfm=kr;
     repeated VISIT/ sub = USUBJID type = un;
     contrast 'Week 2 Effect' TRTN -1 1 TRTN*VISIT -1 0 0 0 0 1 0 0 0 0;
     estimate 'Week 2 Effect' TRTN -1 1 TRTN*VISIT -1 0 0 0 0 1 0 0 0 0/
               cl alpha=0.05;
     contrast 'Week 4 Effect' TRTN -1 1 TRTN*VISIT 0 -1 0 0 0 0 1 0 0 0;
     estimate 'Week 4 Effect' TRTN -1 1 TRTN*VISIT 0 -1 0 0 0 0 1 0 0 0/
               cl alpha=0.05;
     contrast 'Week 12 Effect' TRTN -1 1 TRTN*VISIT 0 0 -1 0 0 0 0 1 0 0;
     estimate 'Week 12 Effect' TRTN -1 1 TRTN*VISIT 0 0 -1 0 0 0 0 1 0 0/
               cl alpha=0.05;
     contrast 'Week 24 Effect' TRTN -1 1 TRTN*VISIT 0 0 0 -1 0 0 0 0 1 0;
     estimate 'Week 24 Effect' TRTN -1 1 TRTN*VISIT 0 0 0 -1 0 0 0 0 1 0/
               cl alpha=0.05;
     contrast 'Week 36 Effect' TRTN -1 1 TRTN*VISIT 0 0 0 0 -1 0 0 0 0 1;
     estimate 'Week 36 Effect' TRTN -1 1 TRTN*VISIT 0 0 0 0 -1 0 0 0 0 1/
               cl alpha=0.05;
     lsmeans TRTN*VISIT / pdiff cl;
     ods output mixed.LSMeans=LSMeans;
     ods output mixed.Contrasts=Contrasts;
     ods output mixed.Estimates=Estimates;
run;
```

Docusign Envelope ID: E39561FE-8723-4250-B198-4822CBF9C70B

The sample SAS code for the comparison of eGFR between each individual dose versus placebo and between 150 mg versus 75 mg at Weeks 2, 4, 12, 24 and 36 is provided below:

```
**************************************************************************
* TRTN = Treatment group [1=Atacicept 150 mg, 2 =Atacicept 75 mg,
                          3 = Atacicept 25 mg, 4=Placebo]
* USUBJID = Subject ID
* VISIT = Post-baseline study visits
* LOGBASE = log (baseline GFR)
* LOG_CHG = Change of logarithmic GFR at each post-baseline visit
calculated
           as log(AVAL)-LOGBASE where AVAL=GFR value at each visit
**************************************************************************;
proc mixed data=efficacy (where=(LOG_CHG ne . and
          VISIT in ('Week 2', 'Week 4', 'Week 12', 'Week 24', 'Week
36')))
           method=reml;
     class TRTN USUBJID VISIT;
     model LOG_CHG = TRTN VISIT LOGBASE TRTN*VISIT /ddfm=kr;
     repeated VISIT/ sub = USUBJID type = un;
     contrast 'Week 2 Effect 150 mg vs. Placebo'
        TRTN 1 0 0 -1 TRTN*VISIT 1 0 0 0 0 0 0 0 0 0 0 0 0 0 0 -1 0 0 0 0;
     contrast 'Week 2 Effect 75 mg vs. Placebo'
        TRTN 0 1 0 -1 TRTN*VISIT 0 0 0 0 0 1 0 0 0 0 0 0 0 0 0 -1 0 0 0 0;
     contrast 'Week 2 Effect 25 mg vs. Placebo'
        TRTN 0 0 1 -1 TRTN*VISIT 0 0 0 0 0 0 0 0 0 0 1 0 0 0 0 -1 0 0 0 0;
     contrast 'Week 2 Effect 150 mg vs. 75mg'
        TRTN 1 -1 0 0 TRTN*VISIT 1 0 0 0 0 -1 0 0 0 0 0 0 0 0 0 0 0 0 0 0;

     contrast 'Week 4 Effect 150 mg vs. Placebo'
        TRTN 1 0 0 -1 TRTN*VISIT 0 1 0 0 0 0 0 0 0 0 0 0 0 0 0 0 -1 0 0 0;
     contrast 'Week 4 Effect 75 mg vs. Placebo'
        TRTN 0 1 0 -1 TRTN*VISIT 0 0 0 0 0 0 1 0 0 0 0 0 0 0 0 0 -1 0 0 0;
     contrast 'Week 4 Effect 25 mg vs. Placebo'
        TRTN 0 0 1 -1 TRTN*VISIT 0 0 0 0 0 0 0 0 0 0 0 1 0 0 0 0 -1 0 0 0;
     contrast 'Week 4 Effect 150 mg vs. 75mg'
       TRTN 1 -1 0 0 TRTN*VISIT 0 1 0 0 0 0 -1  0 0 0 0 0 0 0 0 0 0 0 0 0;

     contrast 'Week 12 Effect 150 mg vs. Placebo'
        TRTN 1 0 0 -1 TRTN*VISIT 0 0 1 0 0 0 0 0 0 0 0 0 0 0 0 0 0 -1 0 0;
     contrast 'Week 12 Effect 75 mg vs. Placebo'
        TRTN 0 1 0 -1 TRTN*VISIT 0 0 0 0 0 0 0 1 0 0 0 0 0 0 0 0 0 -1 0 0;
     contrast 'Week 12 Effect 25 mg vs. Placebo'
        TRTN 0 0 1 -1 TRTN*VISIT 0 0 0 0 0 0 0 0 0 0 0 0 1 0 0 0 0 -1 0 0;
     contrast 'Week 12 Effect 150 mg vs. 75mg'
        TRTN 1 -1 0 0 TRTN*VISIT 0 0 1 0 0 0 0 -1 0 0 0 0 0 0 0 0 0 0 0 0;

     contrast 'Week 24 Effect 150 mg vs. Placebo'
        TRTN 1 0 0 -1 TRTN*VISIT 0 0 0 1 0 0 0 0 0 0 0 0 0 0 0 0 0 0 -1 0;
     contrast 'Week 24 Effect 75 mg vs. Placebo'
        TRTN 0 1 0 -1 TRTN*VISIT 0 0 0 0 0 0 0 0 1 0 0 0 0 0 0 0 0 0 -1 0;
     contrast 'Week 24 Effect 25 mg vs. Placebo'
        TRTN 0 0 1 -1 TRTN*VISIT 0 0 0 0 0 0 0 0 0 0 0 0 0 1 0 0 0 0 -1 0;
     contrast 'Week 24 Effect 150 mg vs. 75mg'
        TRTN 1 -1 0 0 TRTN*VISIT 0 0 0 1 0 0 0 0 -1 0 0 0 0 0 0 0 0 0 0 0;
```

Docusign Envelope ID: E39561FE-8723-4250-B198-4822CBF9C70B


```
     contrast 'Week 36 Effect 150 mg vs. Placebo'
        TRTN 1 0 0 -1 TRTN*VISIT 0 0 0 0 1 0 0 0 0 0 0 0 0 0 0 0 0 0 0 -1;
     contrast 'Week 36 Effect 75 mg vs. Placebo'
        TRTN 0 1 0 -1 TRTN*VISIT 0 0 0 0 0 0 0 0 0 1 0 0 0 0 0 0 0 0 0 -1;
     contrast 'Week 36 Effect 25 mg vs. Placebo'
        TRTN 0 0 1 -1 TRTN*VISIT 0 0 0 0 0 0 0 0 0 0 0 0 0 0 1 0 0 0 0 -1;
     contrast 'Week 36 Effect 150 mg vs. 75mg'
        TRTN 1 -1 0 0 TRTN*VISIT 0 0 0 0 1 0 0 0 0 -1 0 0 0 0 0 0 0 0 0 0;
     lsmeans TRTN*VISIT / pdiff cl;
     ods output mixed.LSMeans=LSMeans;
     ods output mixed.Contrasts=Contrasts;
     ods output mixed.Estimates=Estimates;
run;

Note: estimate statement will be used similarly as contrast statement in the
above code
```

The sample SAS code for the comparison of Immunoglobins (IgA, IgG, IgM) and Gd-IgA1 between each individual dose versus placebo and between 150 mg versus 75 mg at Week 4, Week 12, Week 24, and Week 36 is provided below:

```
**************************************************************************
* TRTN = Treatment group [1=Atacicept 150 mg, 2 =Atacicept 75 mg,
                          3 = Atacicept 25 mg, 4=Placebo]
* USUBJID = Subject ID
* VISIT = Post-baseline study visits
* GFR = Baseline eGFR category [<45 vs. >=45 mL/min/1.73m2]
* LOGBASE = log (baseline value)
* LOG_CHG = Change of logarithmic value at each post-baseline visit
        calculated as log(AVAL)-LOGBASE where AVAL=lab value at each visit
*************************************************************************;
proc mixed data=efficacy (where=(LOG_CHG ne . and
     VISIT in ('Week 4', 'Week 12', 'Week 24', 'Week 36'))) method=reml;
     class TRTN USUBJID VISIT GFR;
     model LOG_CHG = TRTN VISIT GFR LOGBASE TRTN*VISIT /ddfm=kr;
     repeated VISIT/ sub = USUBJID type = un;
     contrast 'Week 4 Effect 150 mg vs. Placebo'
               TRTN 1 0 0 -1 TRTN*VISIT 1 0 0 0 0 0 0 0 0 0 0 0 -1 0 0 0;
     contrast 'Week 4 Effect 75 mg vs. Placebo'
               TRTN 0 1 0 -1 TRTN*VISIT 0 0 0 0 1 0 0 0 0 0 0 0 -1 0 0 0;
     contrast 'Week 4 Effect 25 mg vs. Placebo'
               TRTN 0 0 1 -1 TRTN*VISIT 0 0 0 0 0 0 0 0 1 0 0 0 -1 0 0 0;
     contrast 'Week 4 Effect 150 mg vs. 75mg'
               TRTN 1 -1 0 0 TRTN*VISIT 1 0 0 0 -1 0 0 0 0 0 0 0 0 0 0 0;
     contrast 'Week 12 Effect 150 mg vs. Placebo'
               TRTN 1 0 0 -1 TRTN*VISIT 0 1 0 0 0 0 0 0 0 0 0 0 0 -1 0 0;
     contrast 'Week 12 Effect 75 mg vs. Placebo'
               TRTN 0 1 0 -1 TRTN*VISIT 0 0 0 0 0 1 0 0 0 0 0 0 0 -1 0 0;
     contrast 'Week 12 Effect 25 mg vs. Placebo'
               TRTN 0 0 1 -1 TRTN*VISIT 0 0 0 0 0 0 0 0 0 1 0 0 0 -1 0 0;
     contrast 'Week 12 Effect 150 mg vs. 75mg'
               TRTN 1 -1 0 0 TRTN*VISIT 0 1 0 0 0 -1 0 0 0 0 0 0 0 0 0 0;
     contrast 'Week 24 Effect 150 mg vs. Placebo'
               TRTN 1 0 0 -1 TRTN*VISIT 0 0 1 0 0 0 0 0 0 0 0 0 0 0 -1 0;
```

Docusign Envelope ID: E39561FE-8723-4250-B198-4822CBF9C70B


```
     contrast 'Week 24 Effect 75 mg vs. Placebo'
               TRTN 0 1 0 -1 TRTN*VISIT 0 0 0 0 0 0 1 0 0 0 0 0 0 0 -1 0;
     contrast 'Week 24 Effect 25 mg vs. Placebo'
               TRTN 0 0 1 -1 TRTN*VISIT 0 0 0 0 0 0 0 0 0 0 1 0 0 0 -1 0;
     contrast 'Week 24 Effect 150 mg vs. 75mg'
               TRTN 1 -1 0 0 TRTN*VISIT 0 0 1 0 0 0 -1 0 0 0 0 0 0 0 0 0;
     contrast 'Week 36 Effect 150 mg vs. Placebo'
               TRTN 1 0 0 -1 TRTN*VISIT 0 0 0 1 0 0 0 0 0 0 0 0 0 0 0 -1;
     contrast 'Week 36 Effect 75 mg vs. Placebo'
               TRTN 0 1 0 -1 TRTN*VISIT 0 0 0 0 0 0 0 1 0 0 0 0 0 0 0 -1;
     contrast 'Week 36 Effect 25 mg vs. Placebo'
               TRTN 0 0 1 -1 TRTN*VISIT 0 0 0 0 0 0 0 0 0 0 0 1 0 0 0 -1;
     contrast 'Week 36 Effect 150 mg vs. 75mg'
               TRTN 1 -1 0 0 TRTN*VISIT 0 0 0 1 0 0 0 -1 0 0 0 0 0 0 0 0;
     lsmeans TRTN*VISIT / pdiff cl;
     ods output mixed.LSMeans=LSMeans;
     ods output mixed.Contrasts=Contrasts;
     ods output mixed.Estimates=Estimates;
run;

Note: estimate statement will be used similarly as contrast statement in the
above code
```

The sample SAS code for estimating the change from baseline in eGFR using the original scale among the all atacicept and placebo groups is provided below:

```
**************************************************************************
* TRTN = Treatment group [3.75= All Atacicept, 4=Placebo]
* USUBJID = Subject ID
* VISIT = Post-baseline study visits up to Week 96
* BASE = baseline GFR
* CHG = Change of GFR at each post-baseline visit calculated
            as AVAL-BASE where AVAL=GFR value at each visit
*************************************************************************;
proc mixed data=efficacy (where=(CHG ne . and
           VISIT in ('Week 2', 'Week 4', 'Week 12', 'Week 24', ‘Week 36’,
‘Week 38’ ‘Week 40’ ‘Week 48’ ‘Week 60’ ‘Week 72’ ‘Week 84’ ‘Week 96’)))
            method=reml;
     class TRTN USUBJID VISIT;
     model CHG = TRTN VISIT BASE TRTN*VISIT /ddfm=kr;
     repeated VISIT/ sub = USUBJID type = un;
     lsmeans TRTN*VISIT / pdiff cl;
     ods output mixed.LSMeans=LSMeans Diffs = Diffs;
run;
```

The sample SAS code for estimating the annualized total slope in eGFR through Week 96 using the original scale among the all atacicept group is provided below:

```
**************************************************************************
* TRTN = Treatment group [3.75= All Atacicept]
* USUBJID = Subject ID
```

Docusign Envelope ID: E39561FE-8723-4250-B198-4822CBF9C70B


```
* ADYY = Time in year since the first dose of Atacicept (ADYY = 0 for
baseline eGFR).
* BASE = baseline GFR
* AVAL = GFR value using the original scale
************************************************************************;
proc mixed data=efficacy (where=(TRTN =3.75)) method=reml;
     class USUBJID;
     model AVAL = BASE ADYY /ddfm=kr;
     random INT ADYY / sub = USUBJID type = un;
     estimate ‘Slope: All Atacicept’ ADYY 1/cl;
     ods output Estimates = Estimate;
run;
```

Docusign Envelope ID: E39561FE-8723-4250-B198-4822CBF9C70B

Appendix 3: References

Levey AS, Stevens LA, Schmid CH, et al. A new equation to estimate glomerular filtration rate [published correction appears in Ann Intern Med. 2011;155(6):408]. Ann Intern Med. 2009;150(9):604-612.
